# Supplementary material for: General Analysis of Heat Shock Factors in the Cymbidium ensifolium Genome Provided Insights into Their Evolution and Special Roles with Response to Temperature
Source: Int J Mol Sci. 2024 Jan 13;25(2):1002. doi: 10.3390/ijms25021002 (PMC10815800; doi:10.3390/ijms25021002)

**Table S1** Secondary structure of 22 HSF proteins in *C. ensifolium*. Amino acids marked with * every 10 residues.

CeHSF12:


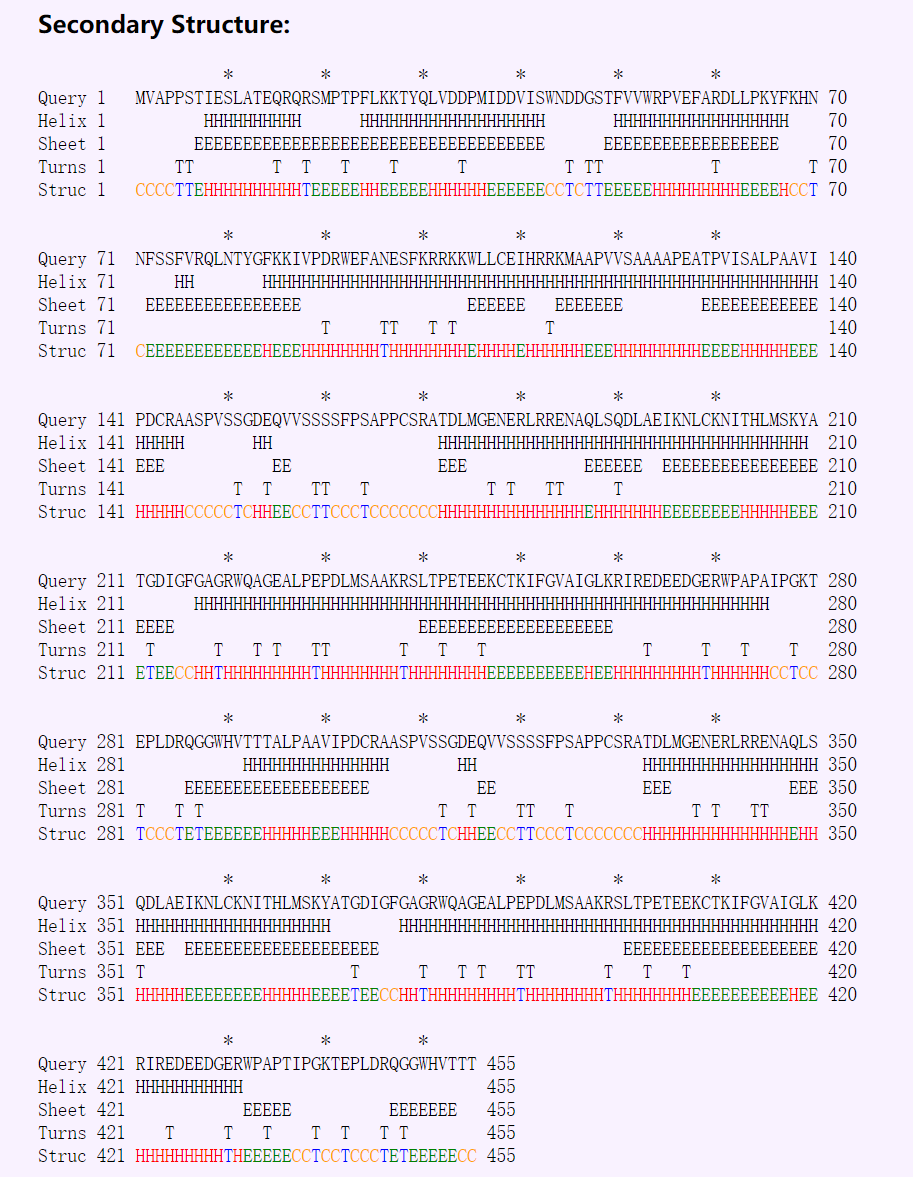


CeHSF8:


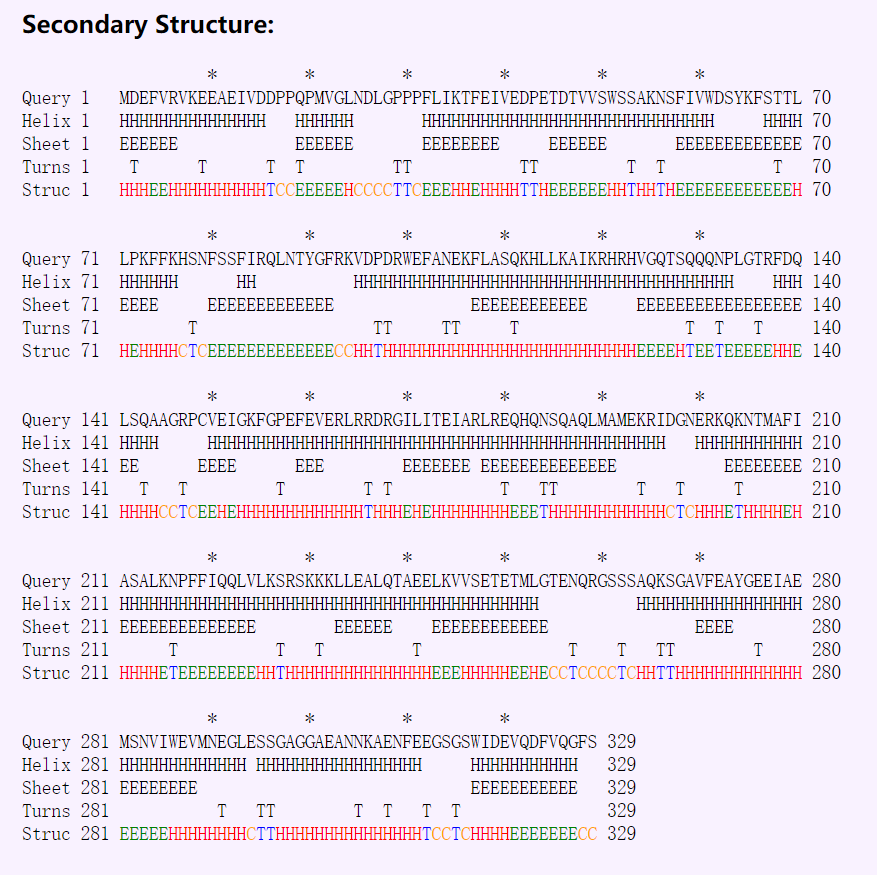


CeHSF16:


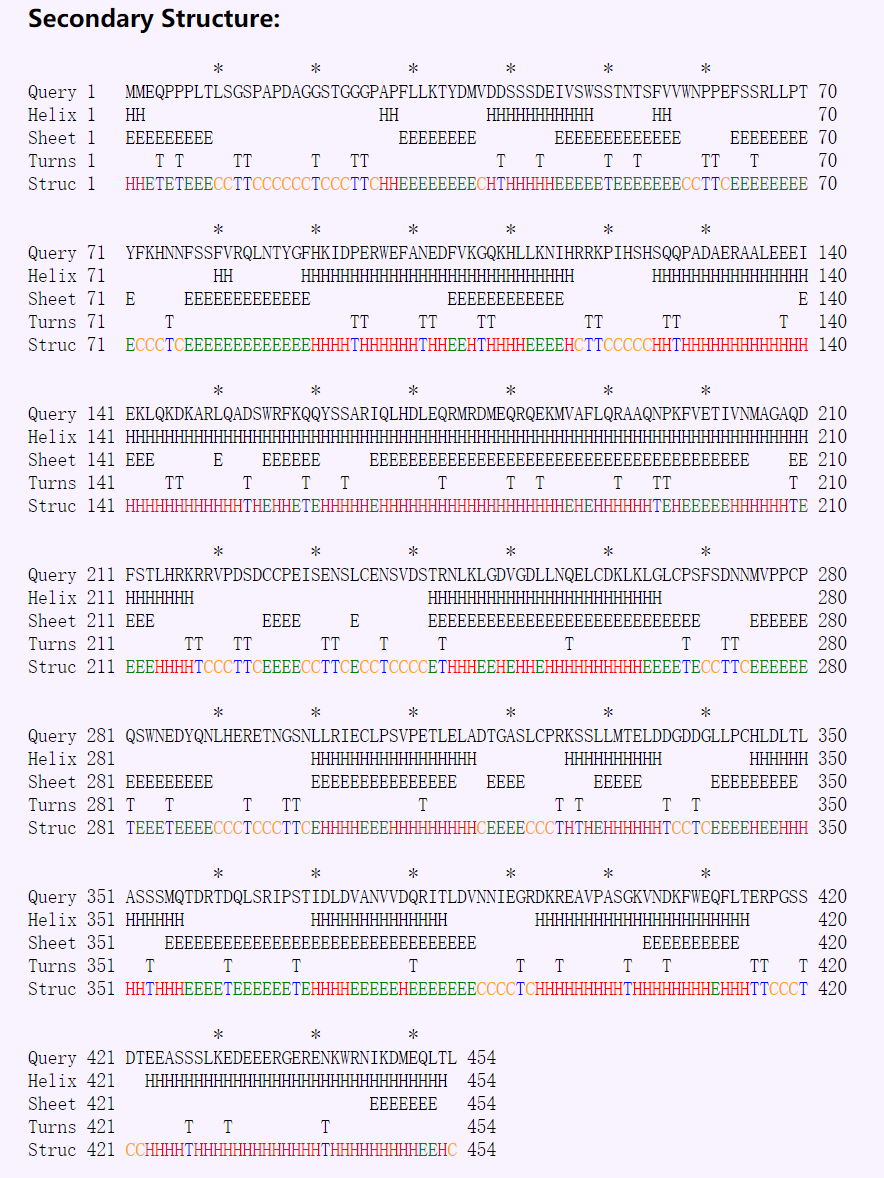


CeHSF21:


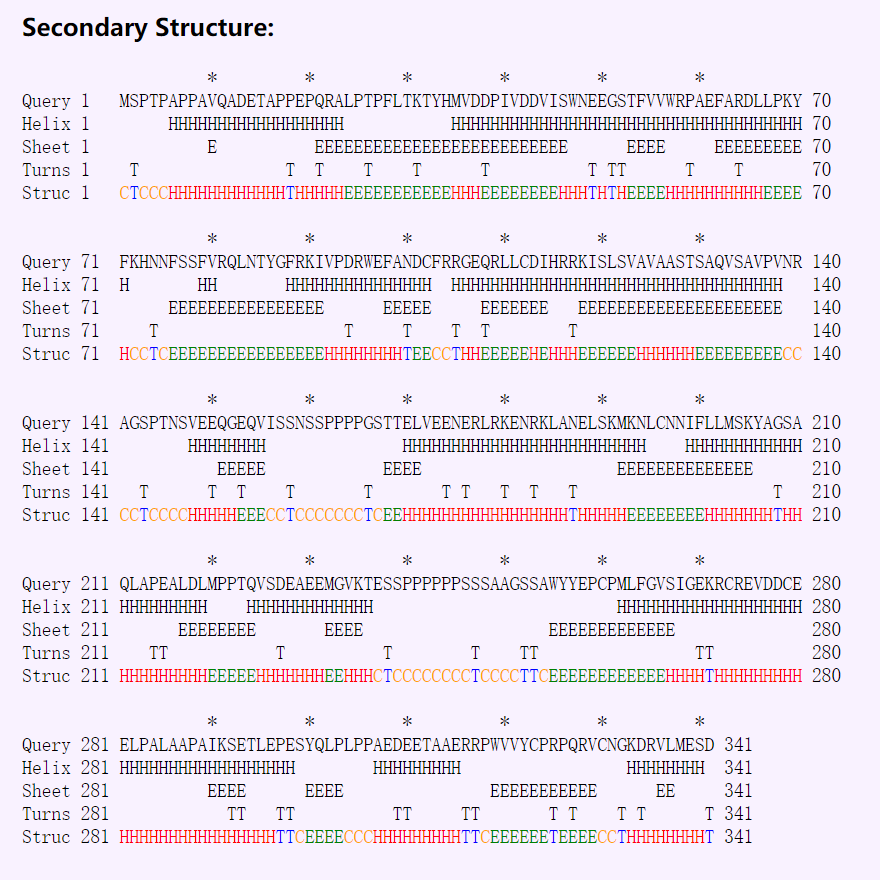


CeHSF6:


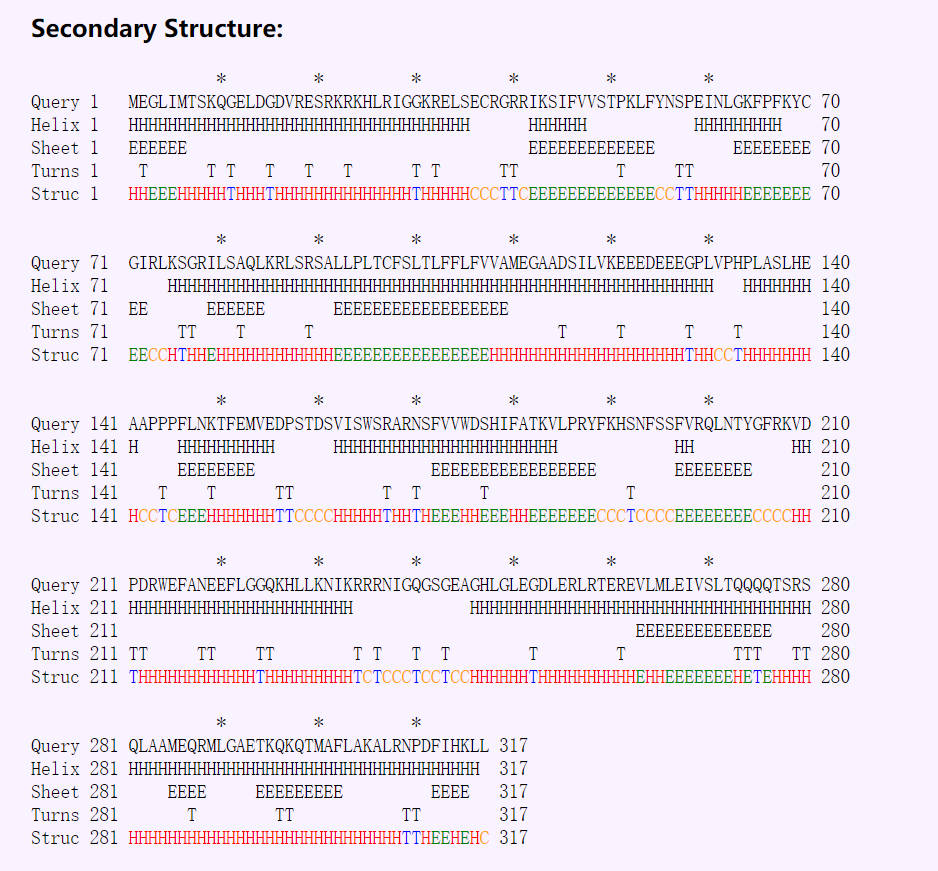


CeHSF5:


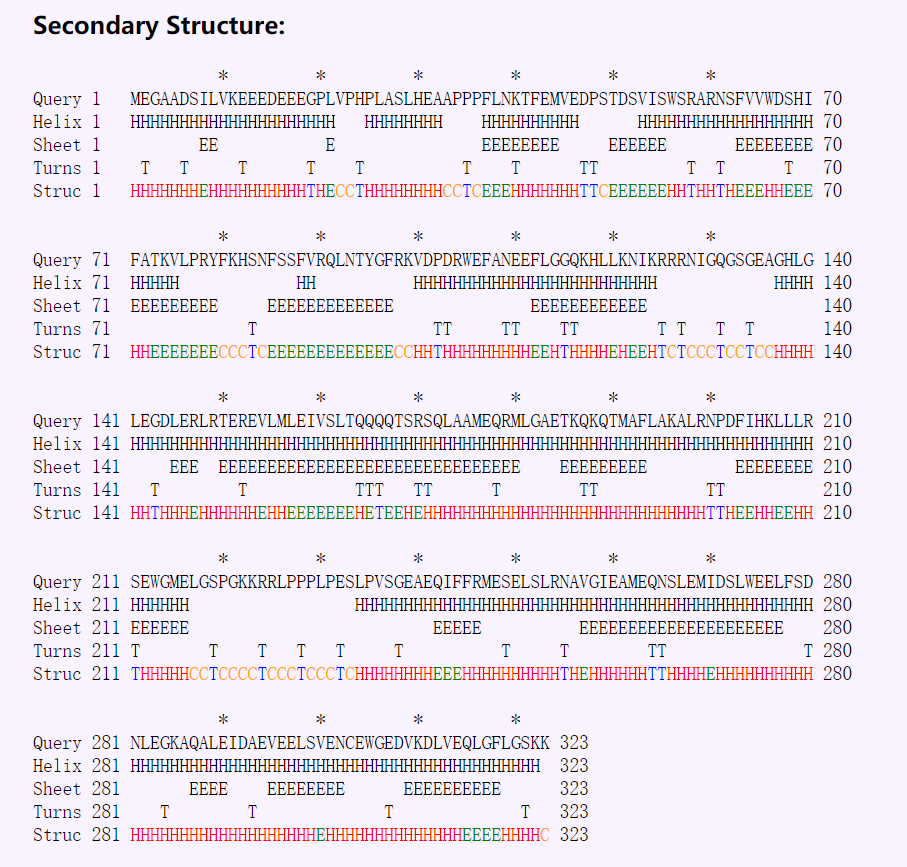


CeHSF4:


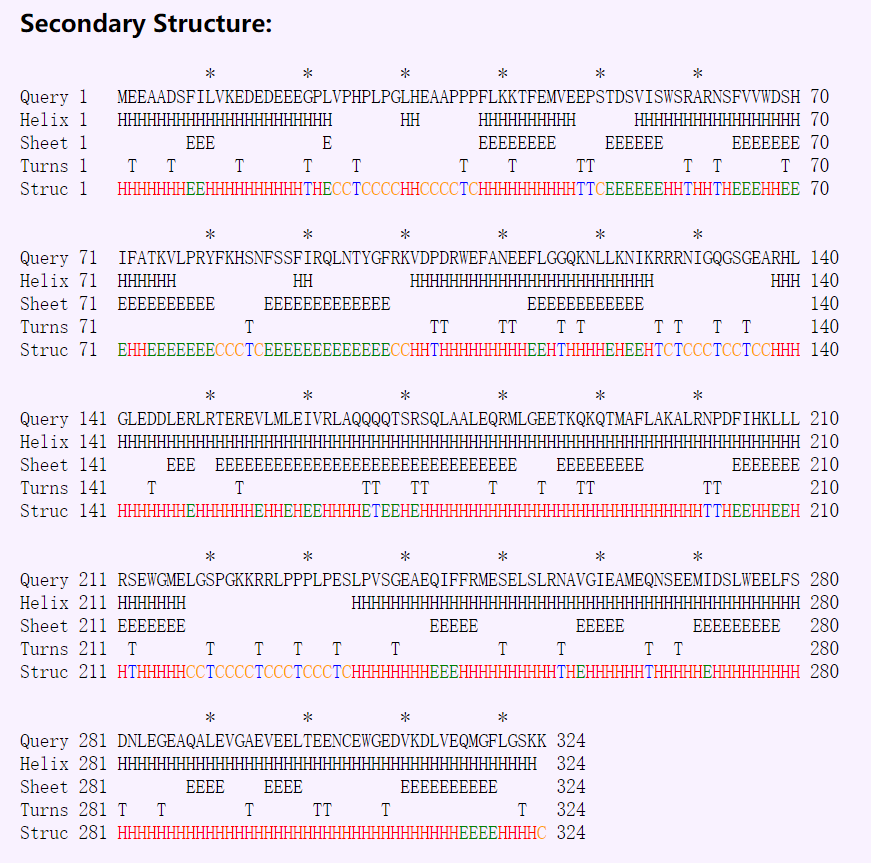


CeHSF7:


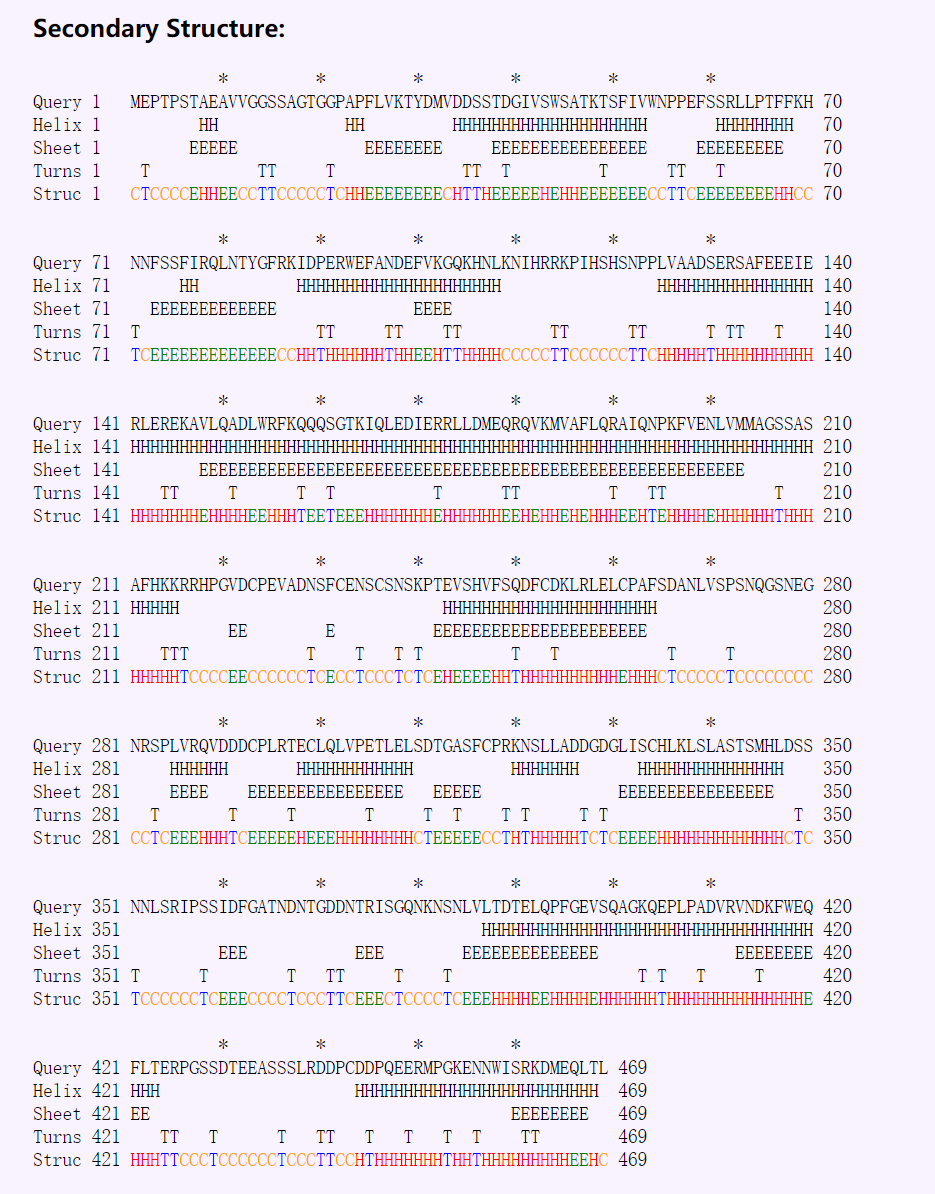


CeHSF19:


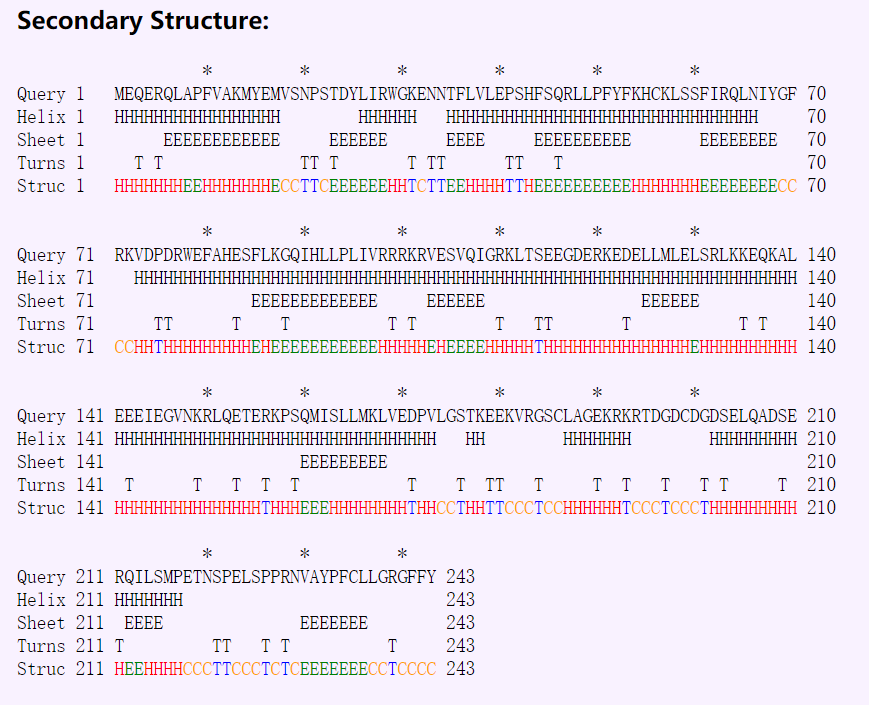


CeHSF15:


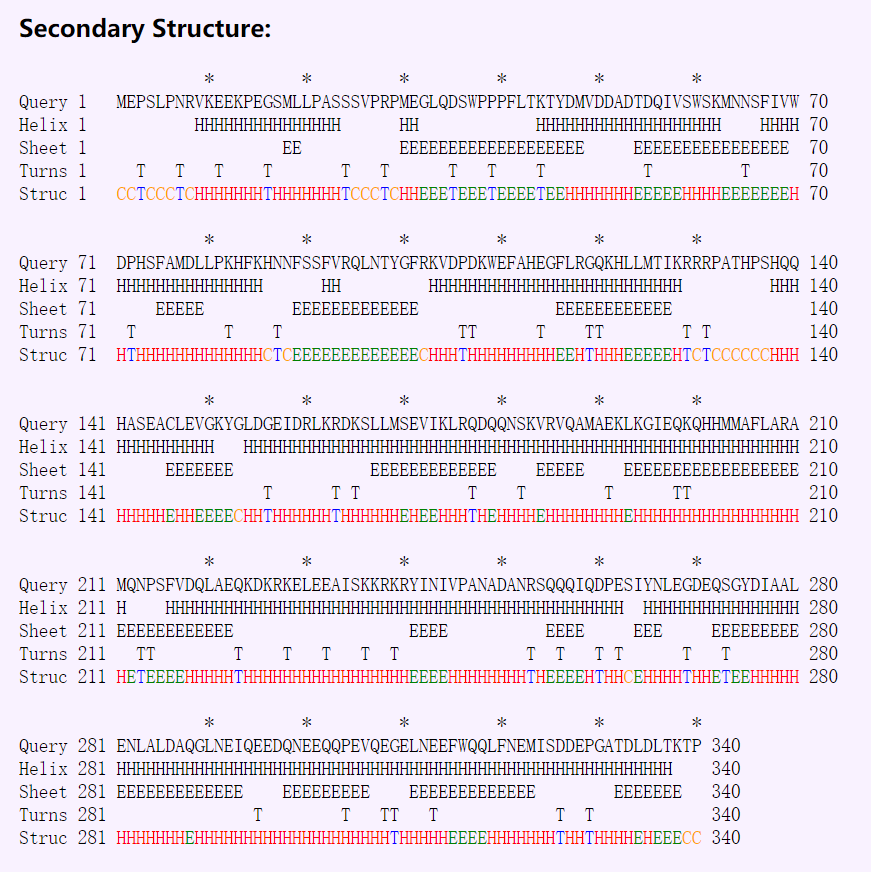


CeHSF9:


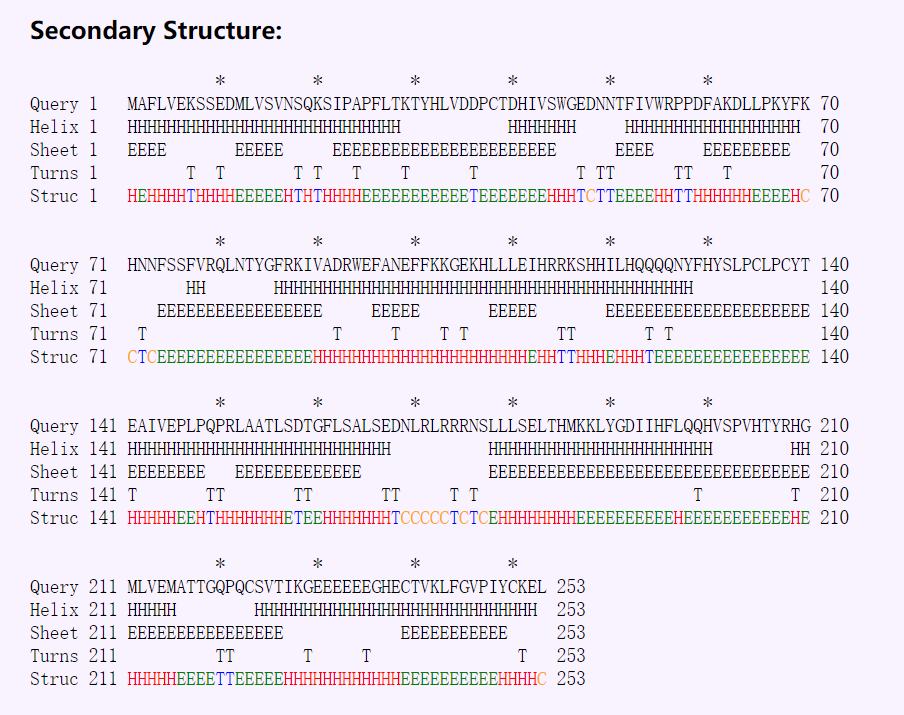


CeHSF14:


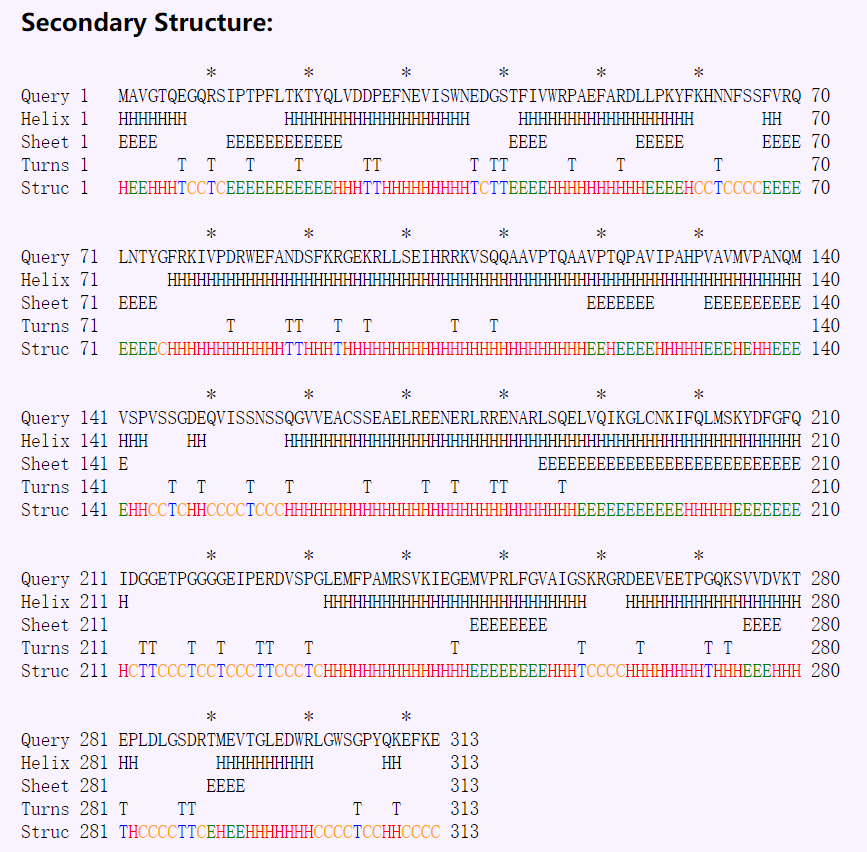


CeHSF17:


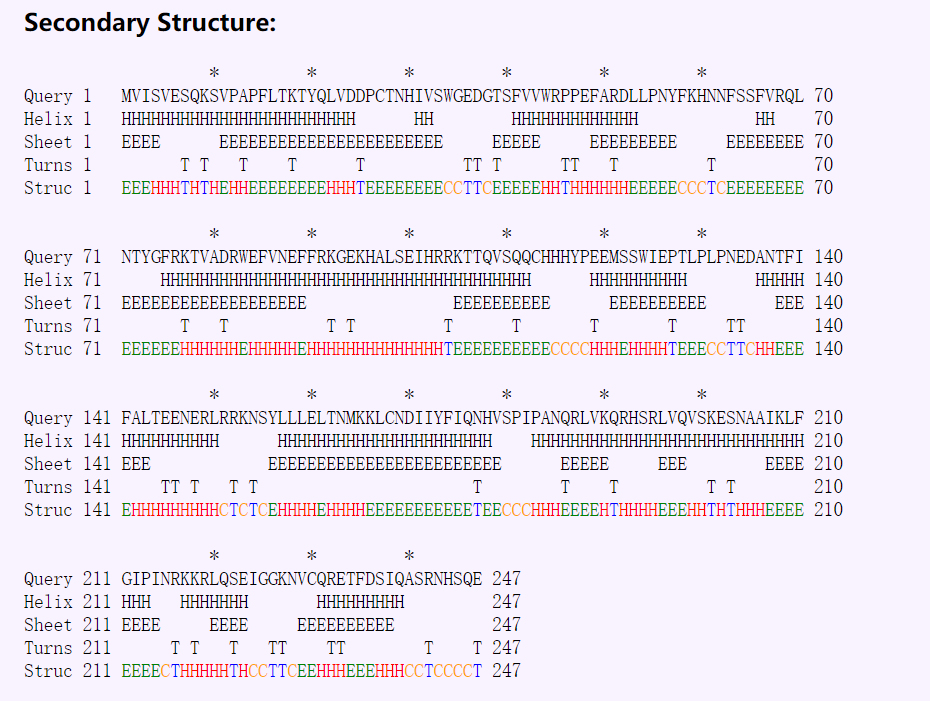


CeHSF18:


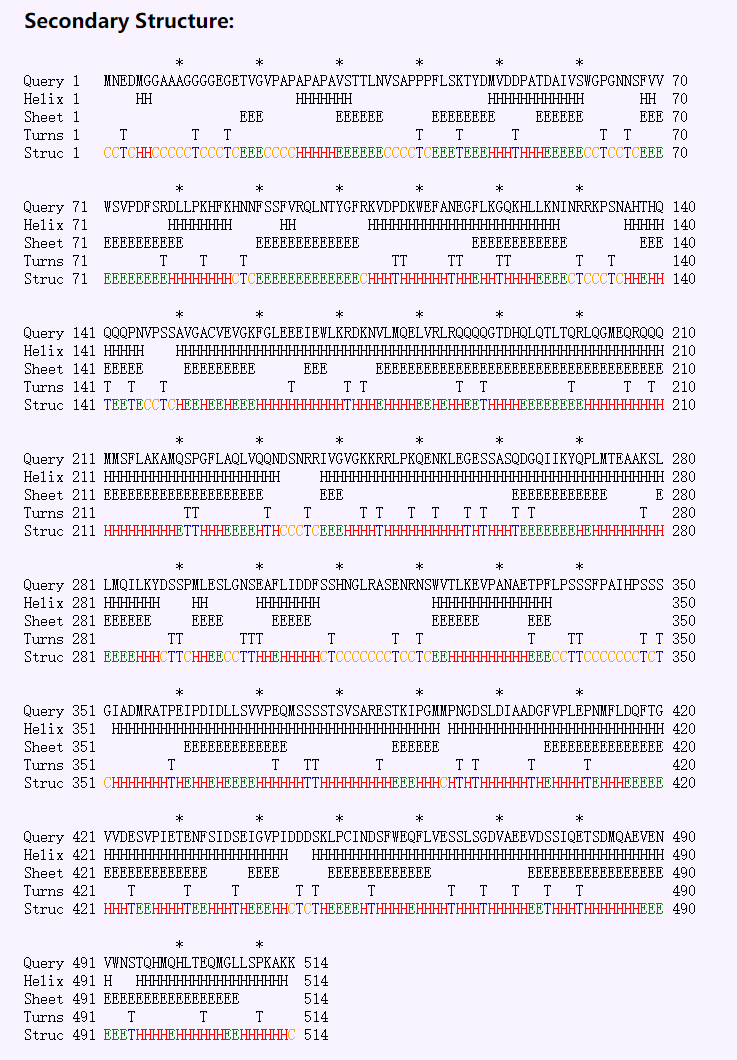


CeHSF13:


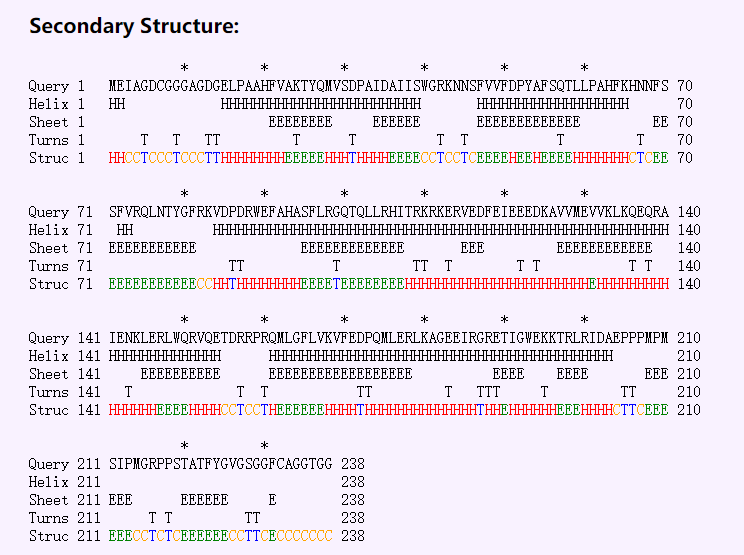


CeHSF22:


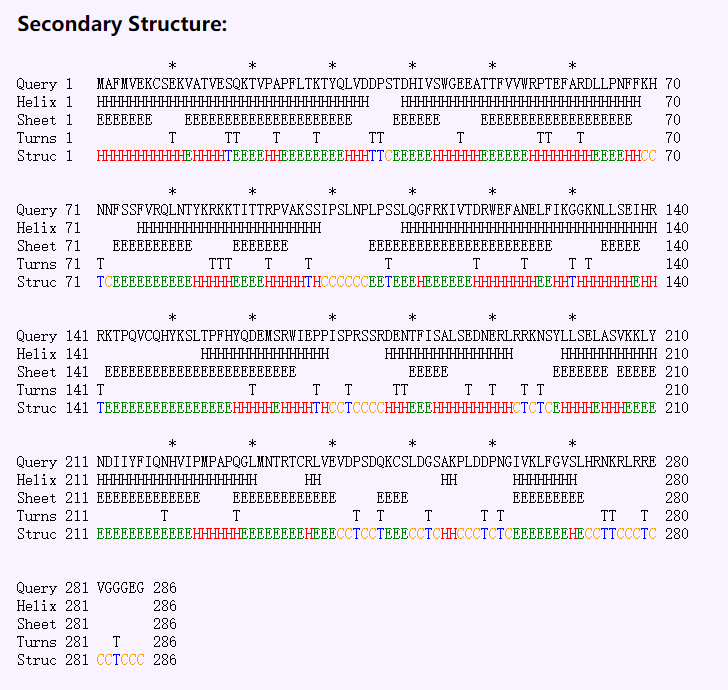


CeHSF11:


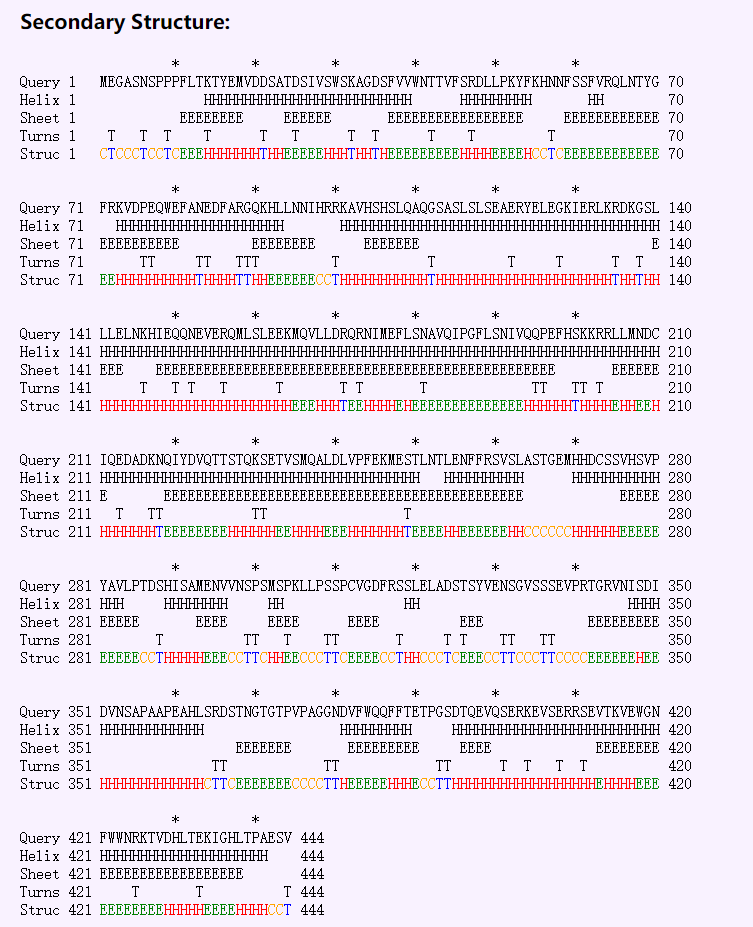


CeHSF20:


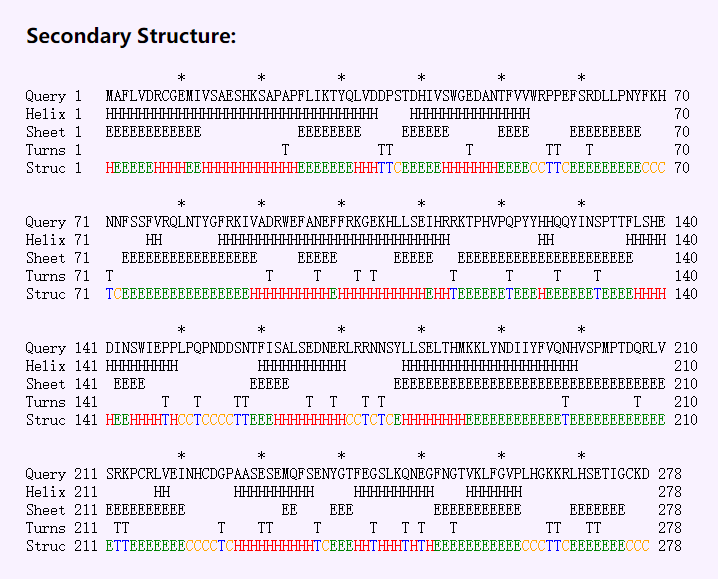


CeHSF10:


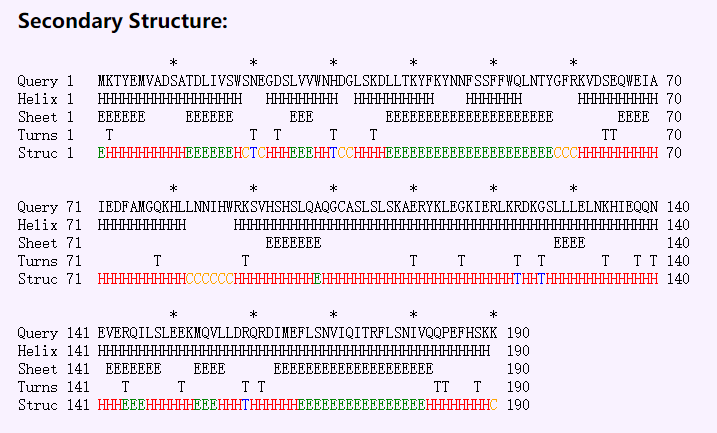


CeHSF3:


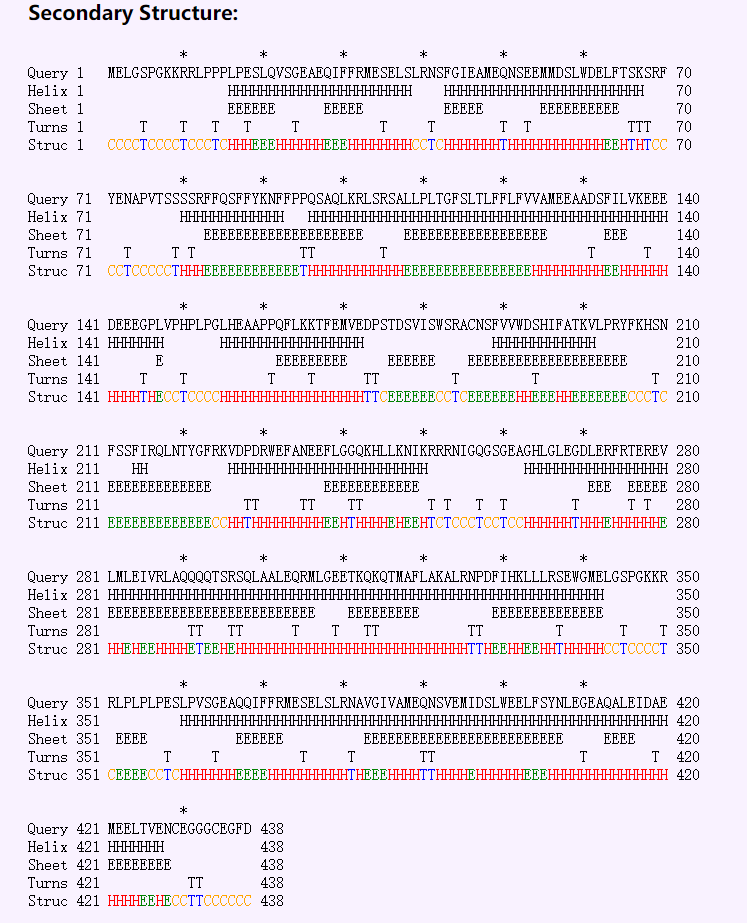


CeHSF2:


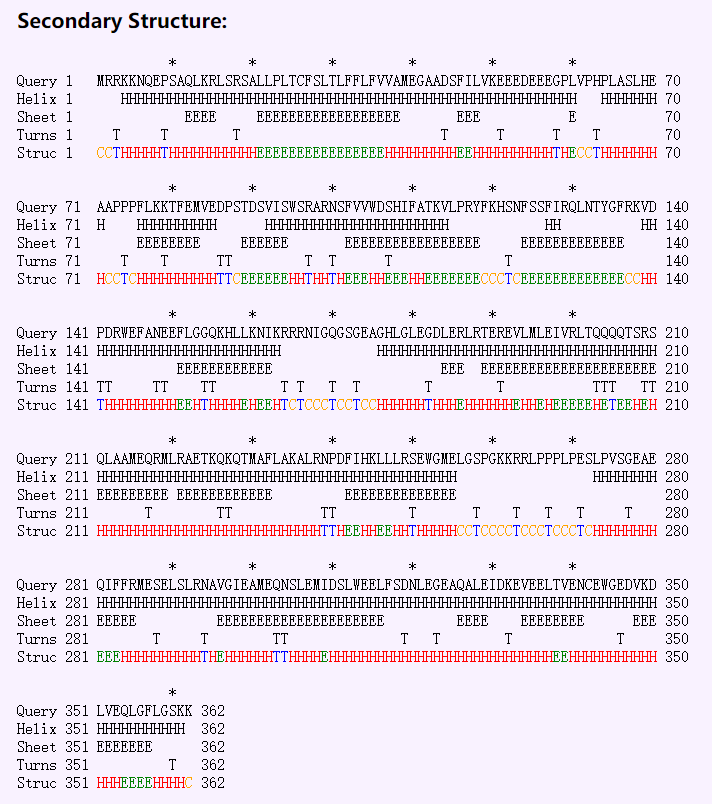


CeHSF1:


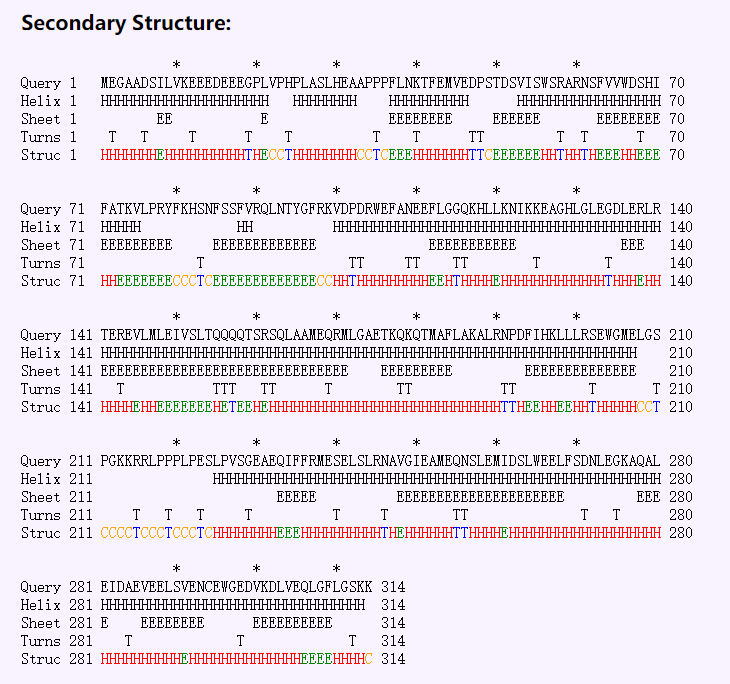

Supplement: Supplementary file 1 [file ijms-25-01002-s001.zip › Supplementary Table S1.docx]
