# Supplementary material for: General Analysis of Heat Shock Factors in the Cymbidium ensifolium Genome Provided Insights into Their Evolution and Special Roles with Response to Temperature
Source: Int J Mol Sci. 2024 Jan 13;25(2):1002. doi: 10.3390/ijms25021002 (PMC10815800; doi:10.3390/ijms25021002)

**Table S2** Tertiary Structure of 22 HSF proteins in *C. ensifolium*.

CeHSF1:


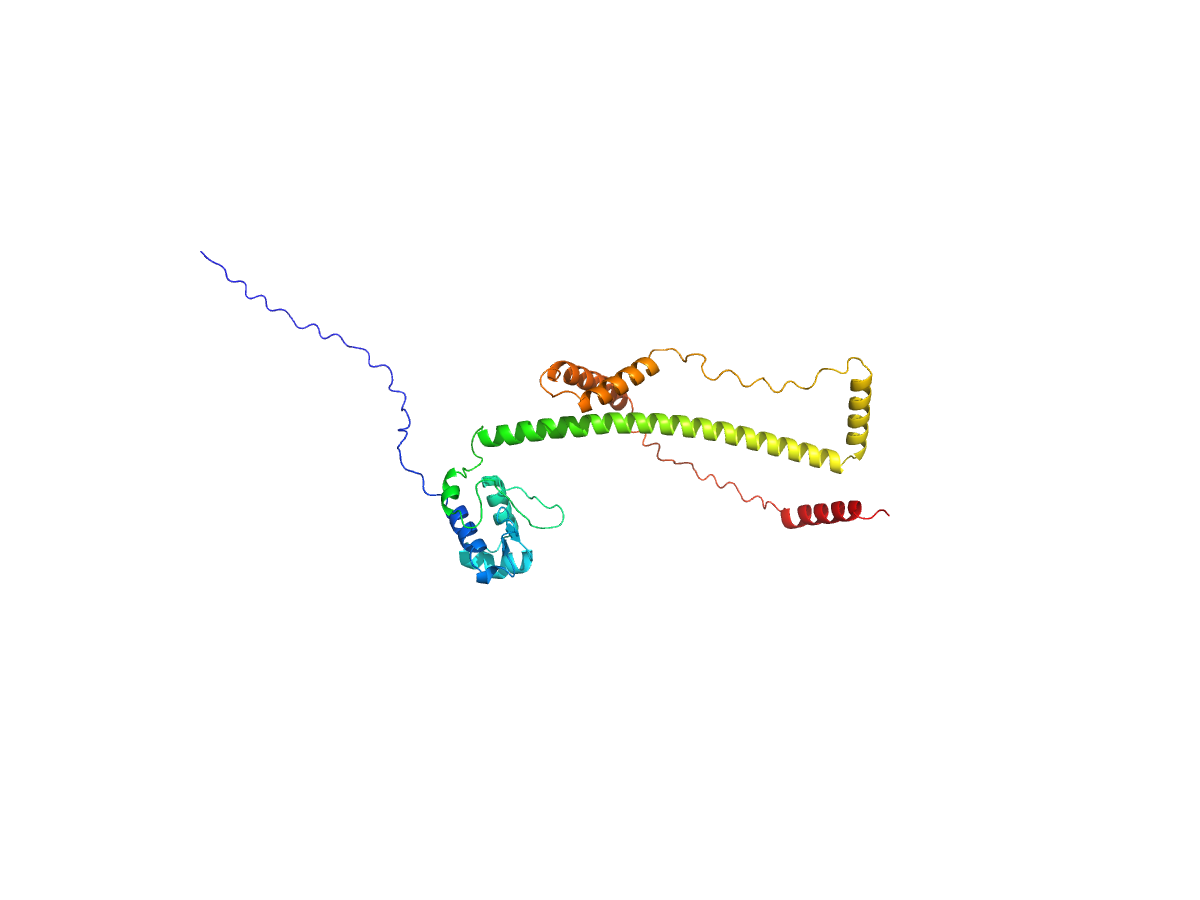


CeHSF2:


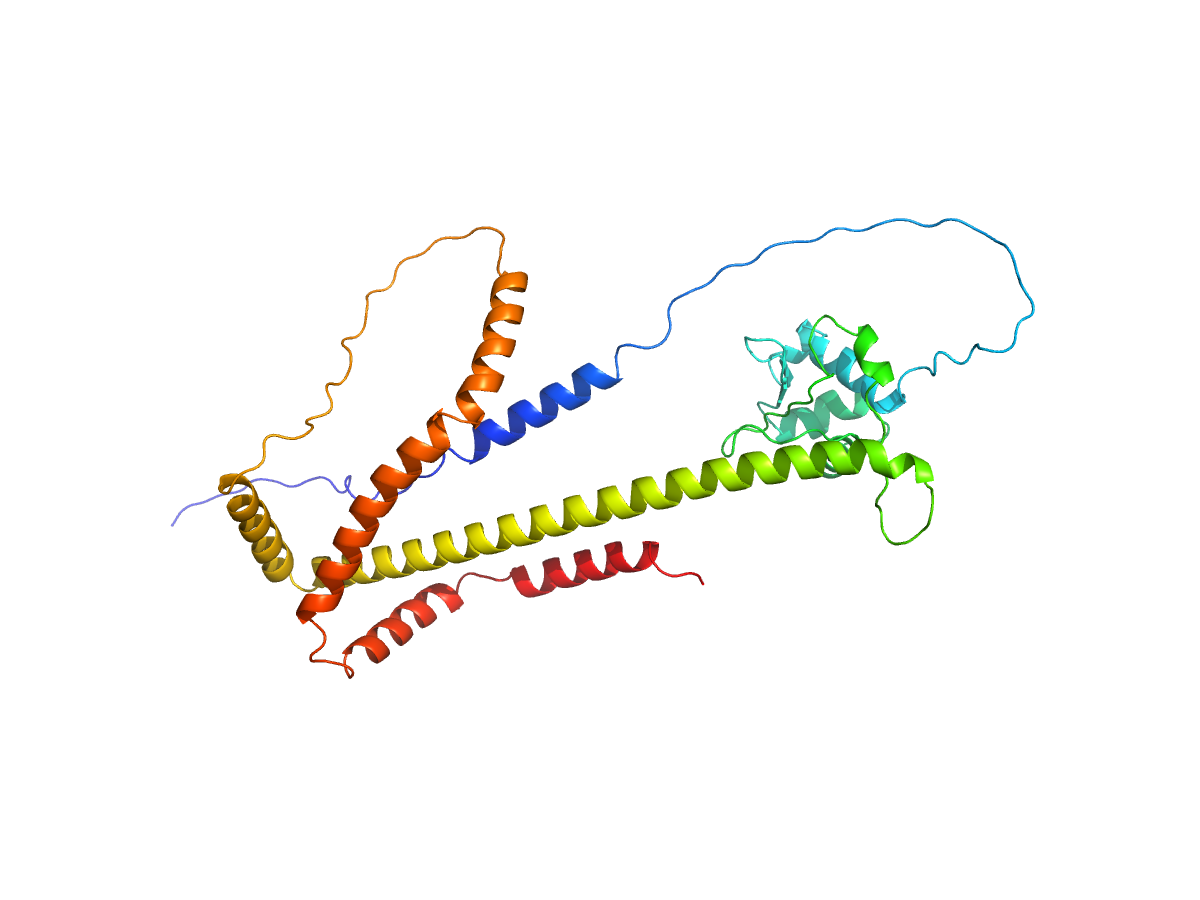


CeHSF3:


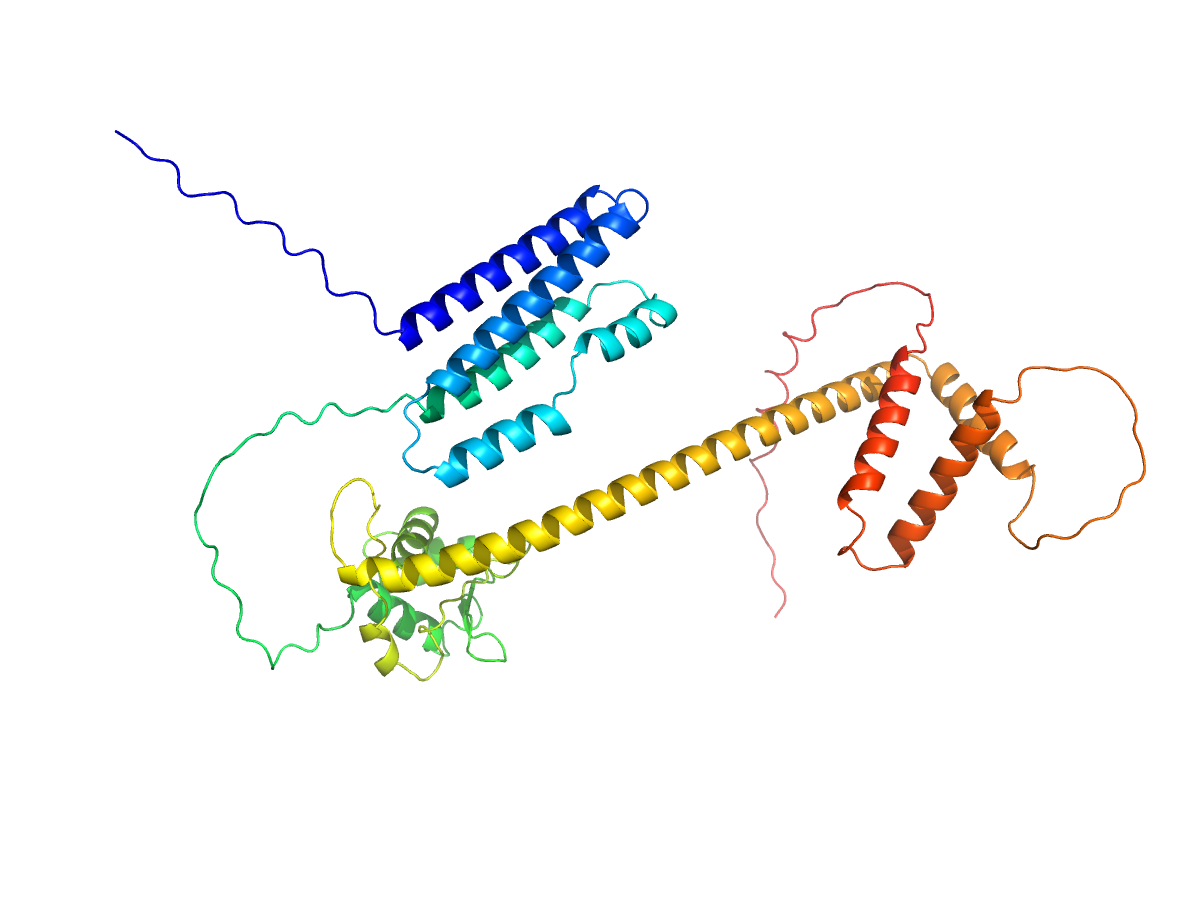


CeHSF4:


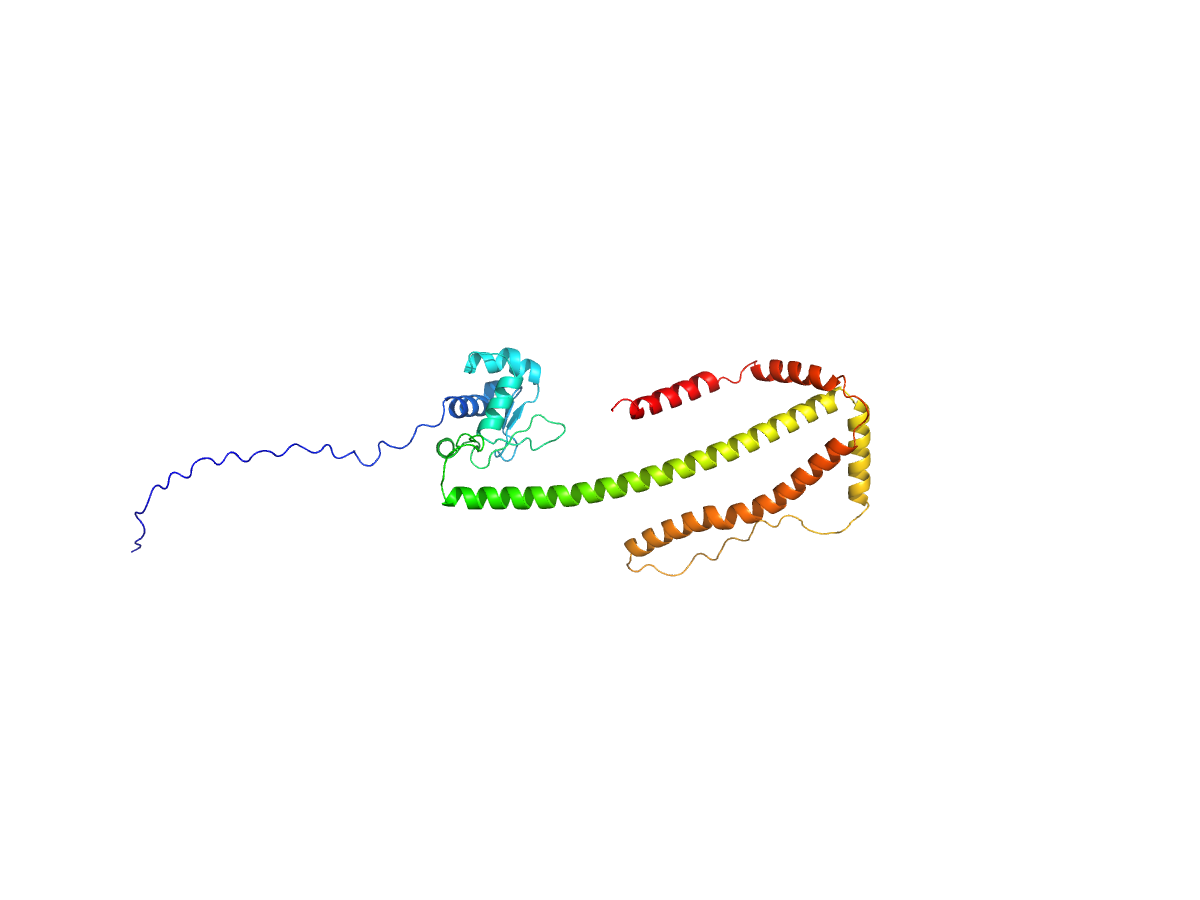


CeHSF5:


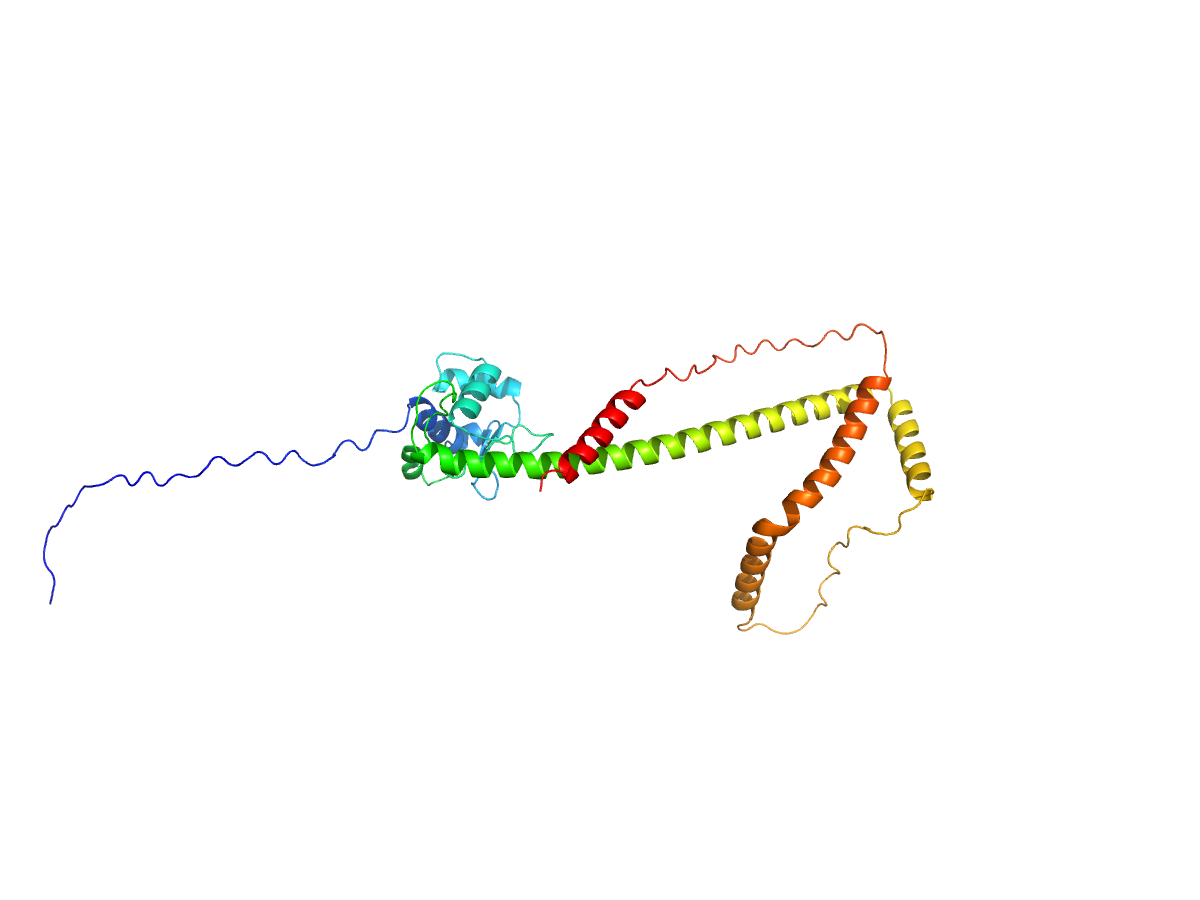


CeHSF6:


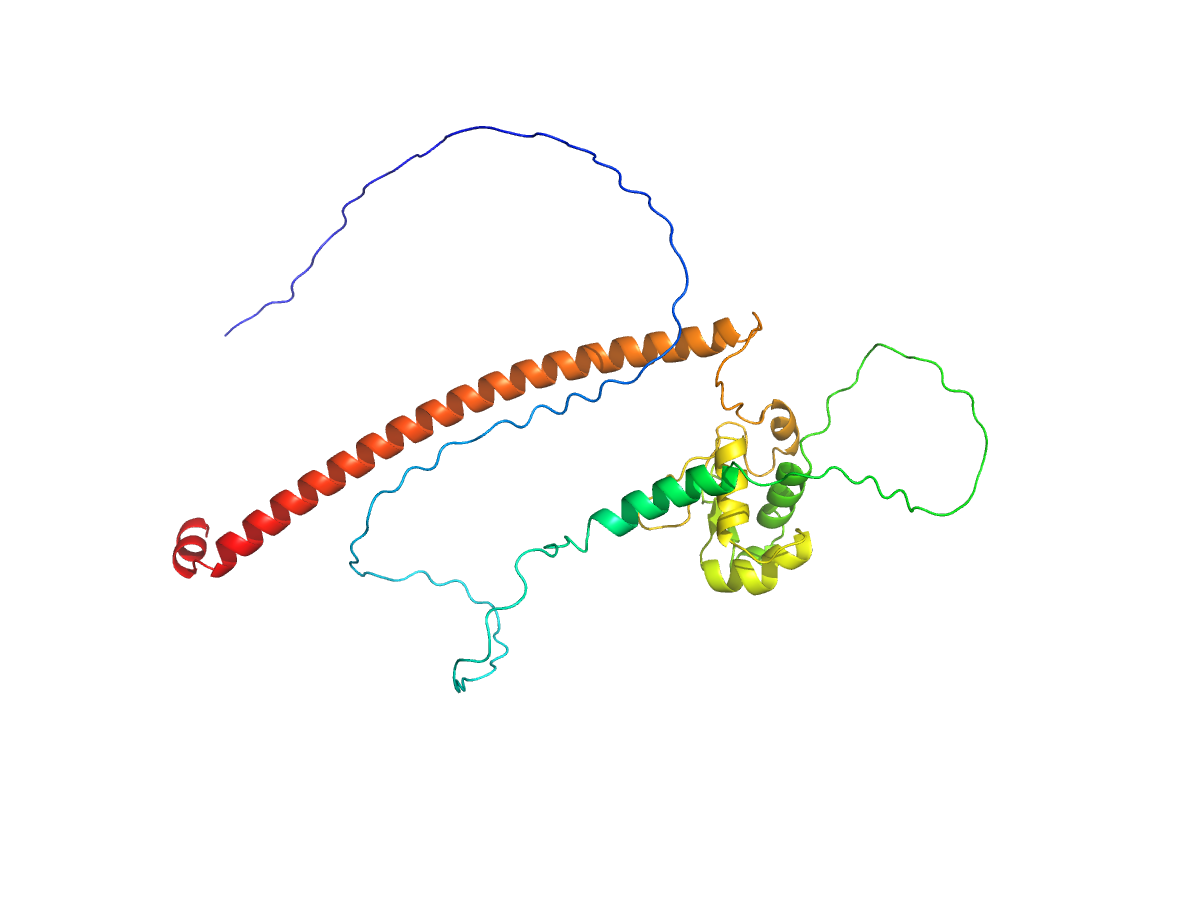


CeHSF7:


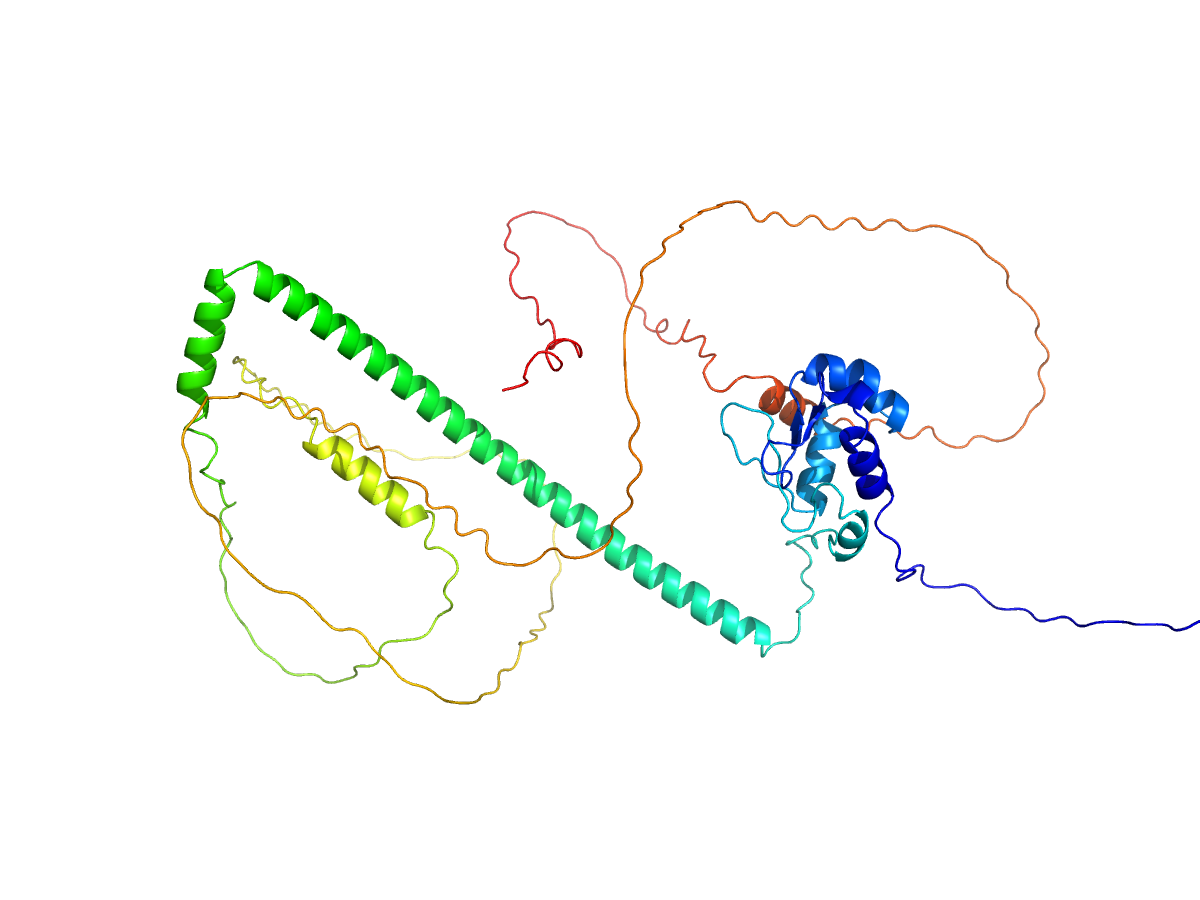


CeHSF8:


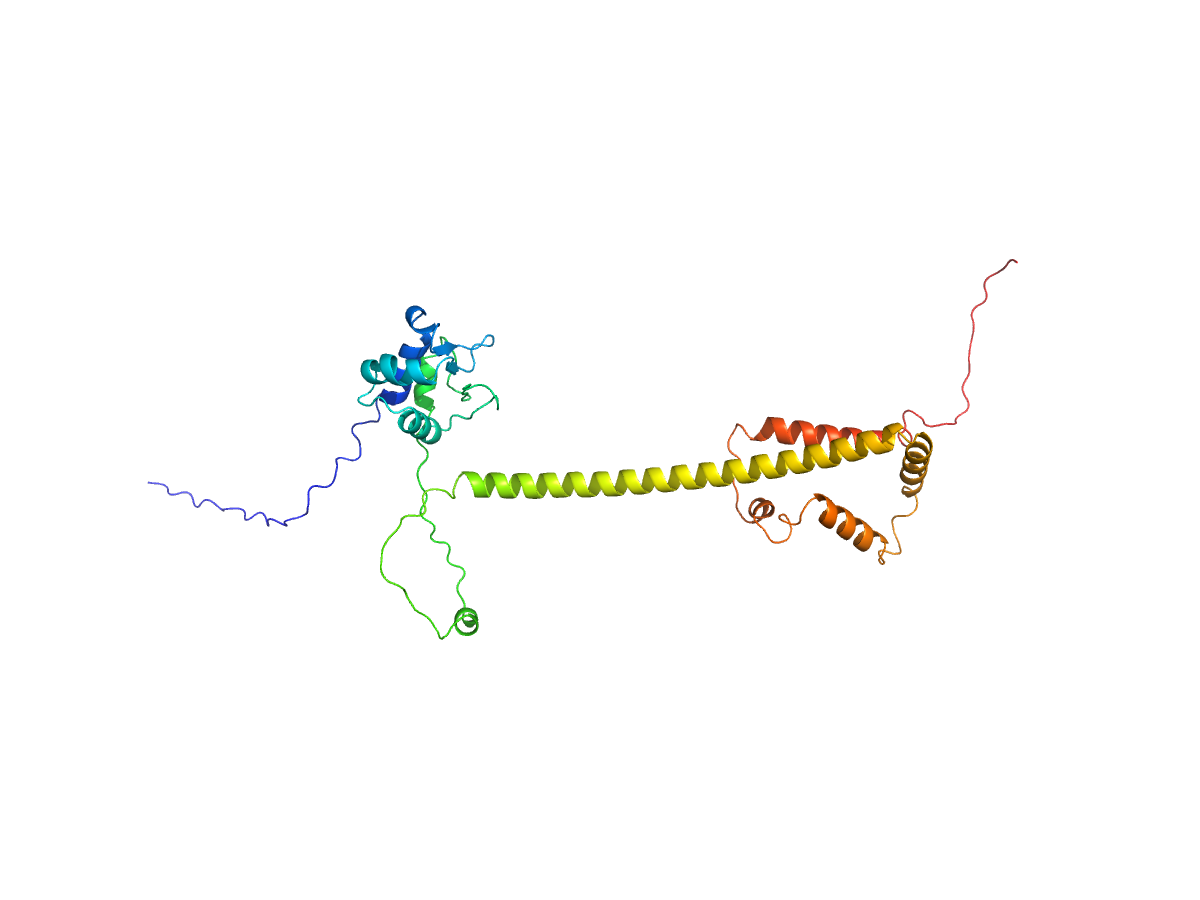


CeHSF9:


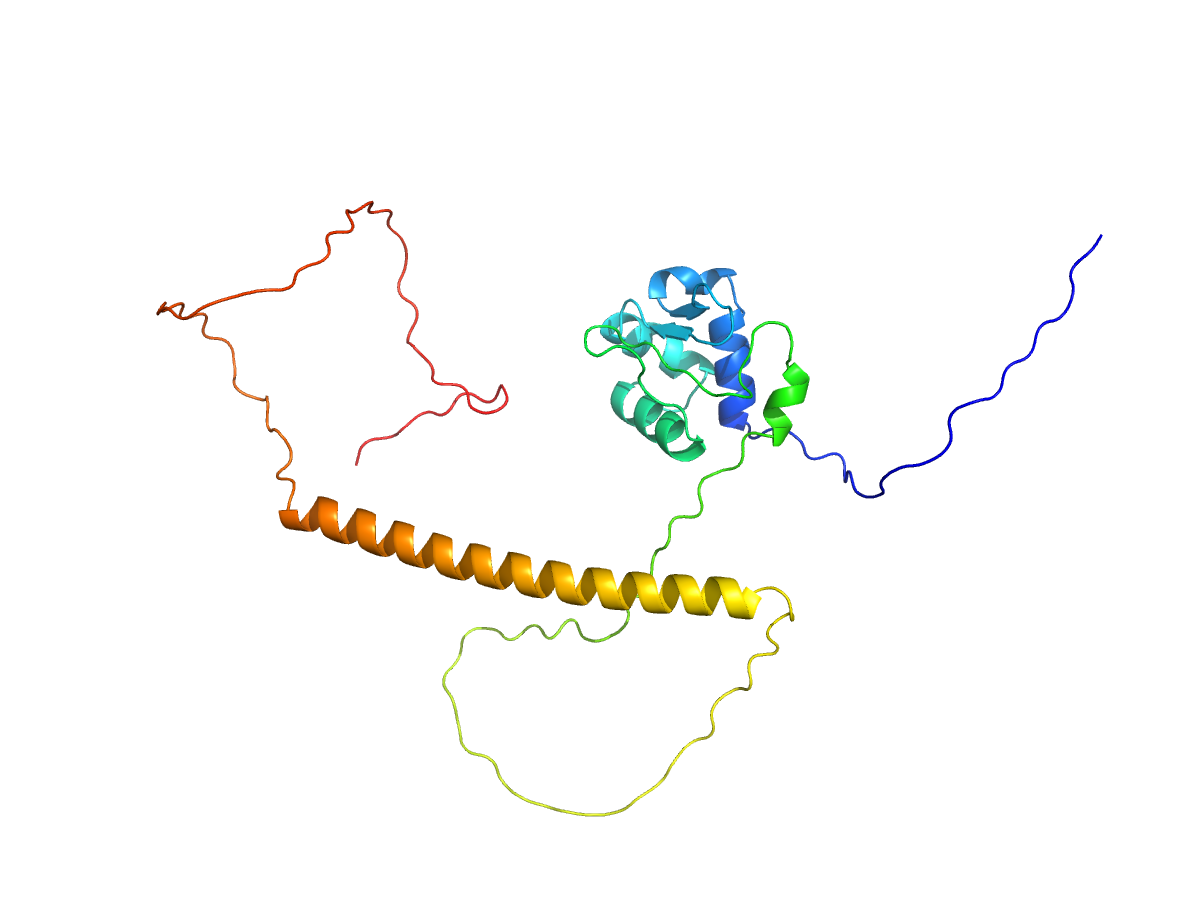


CeHSF10:


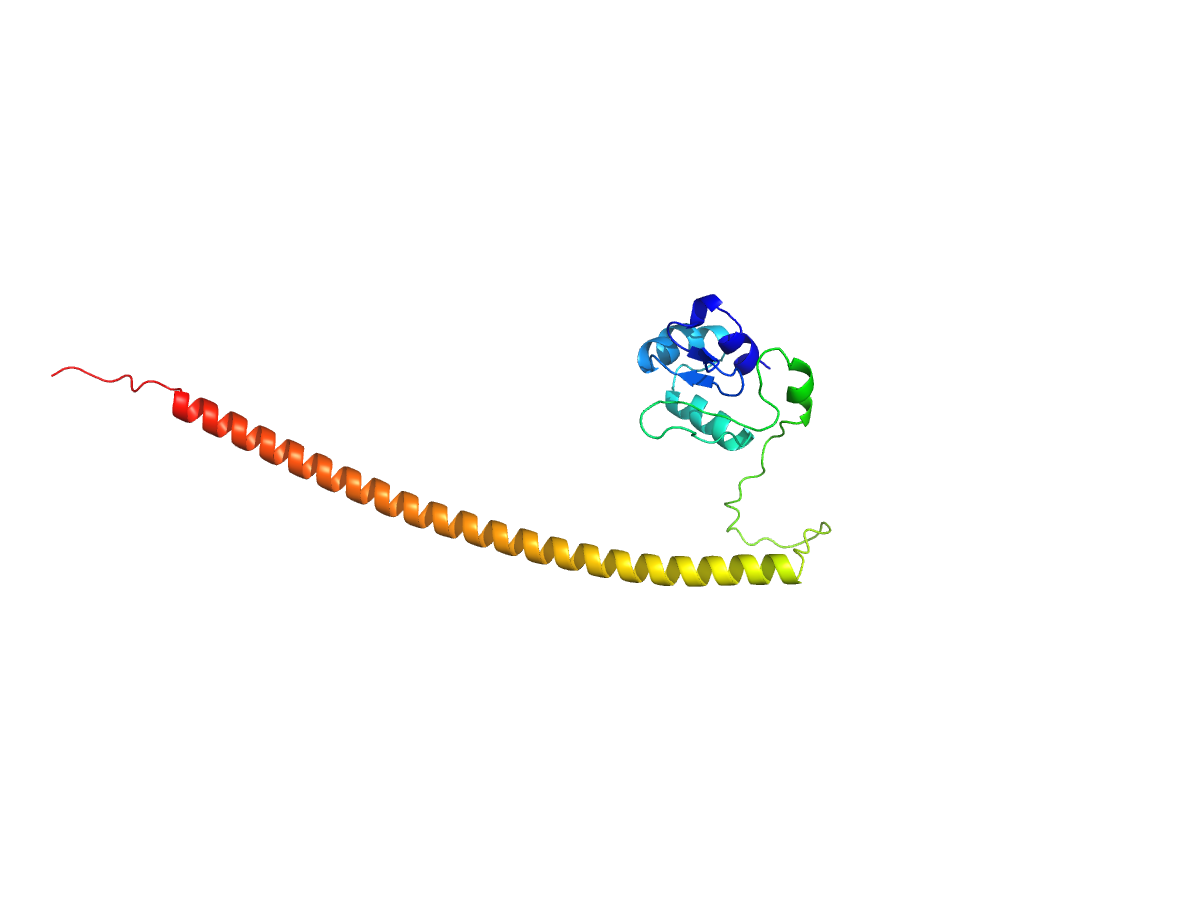


CeHSF11:


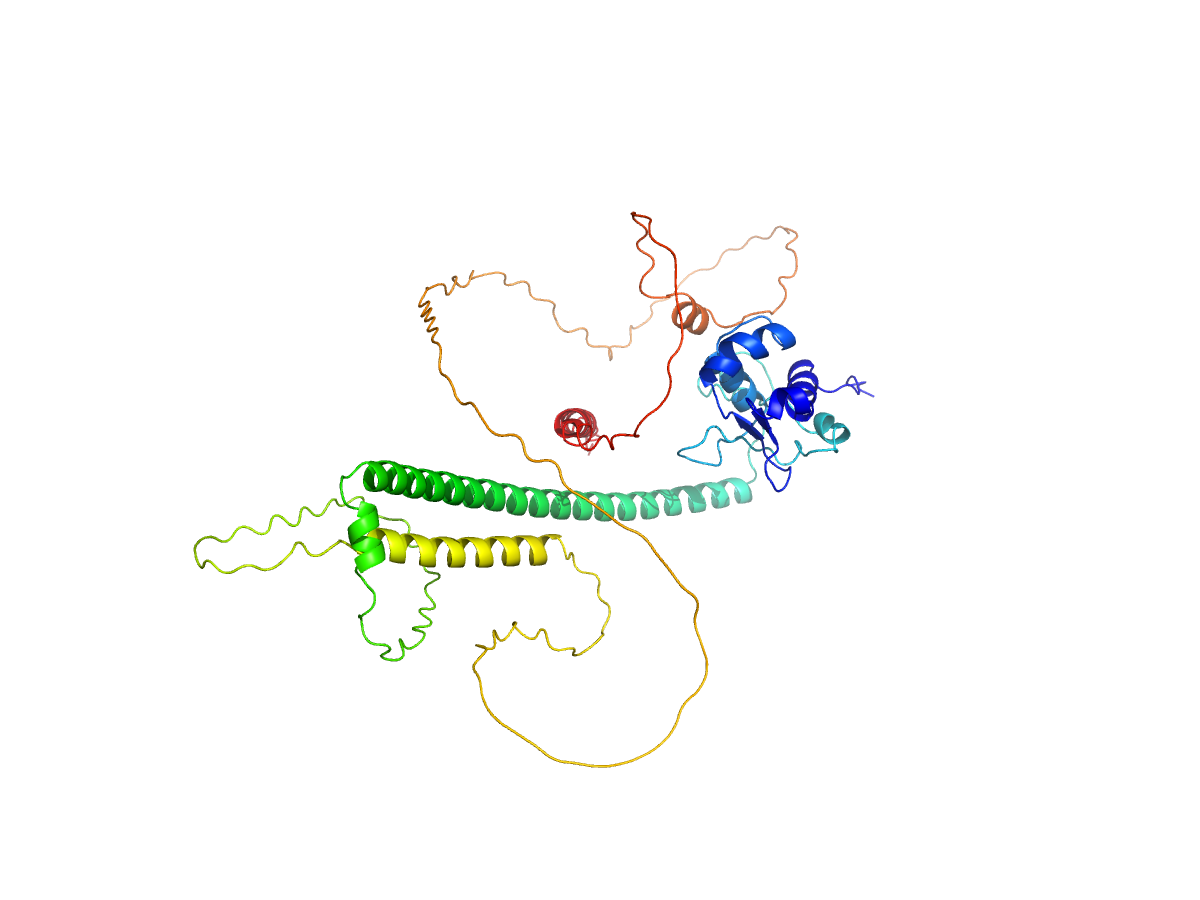


CeHSF12:


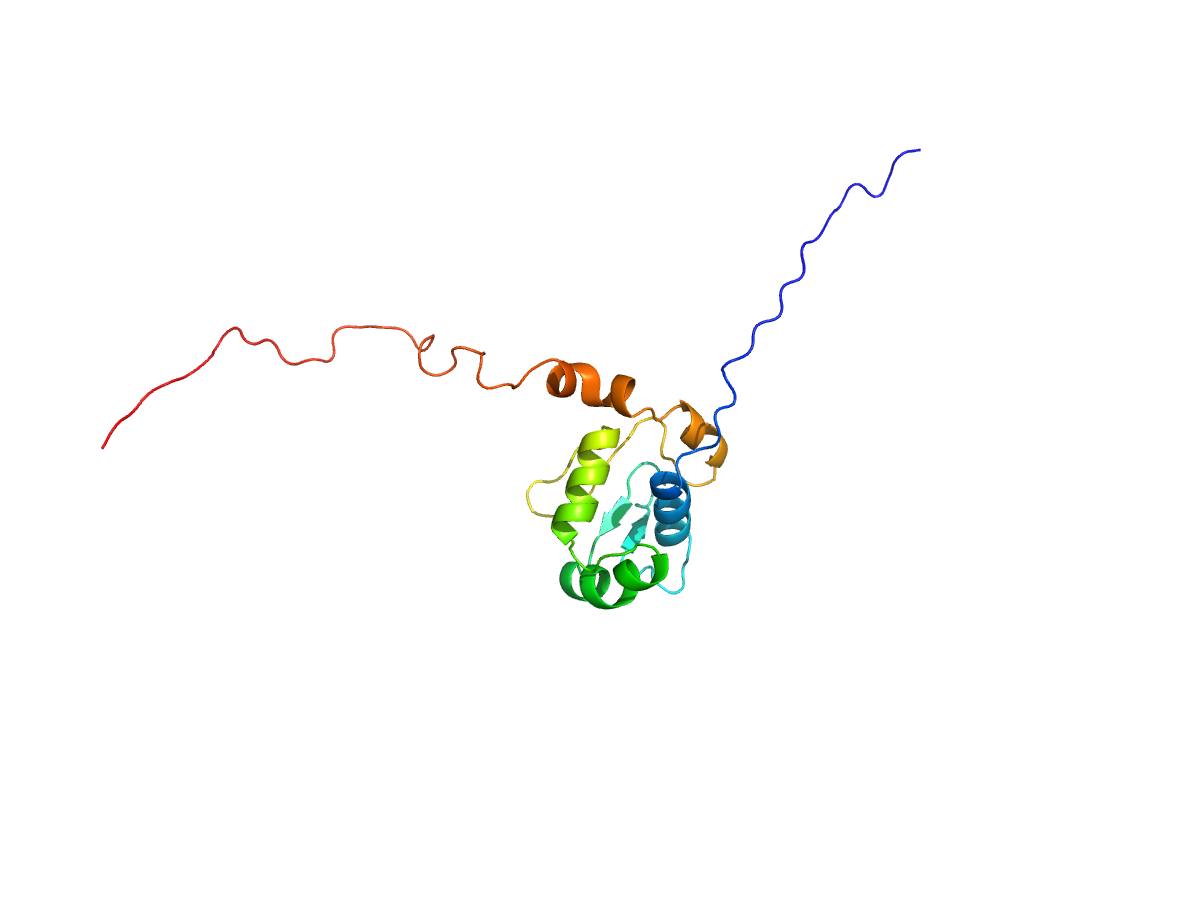


CeHSF13:


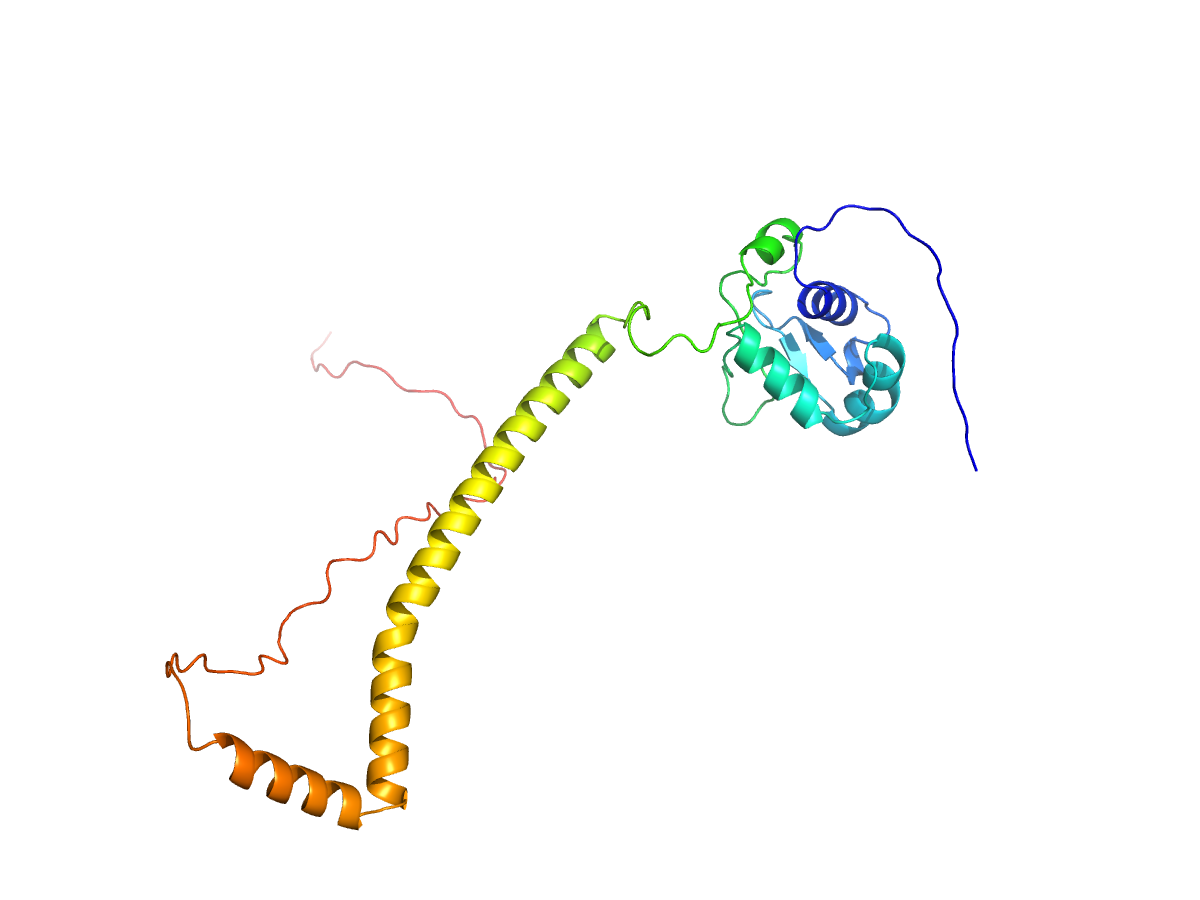


CeHSF14:


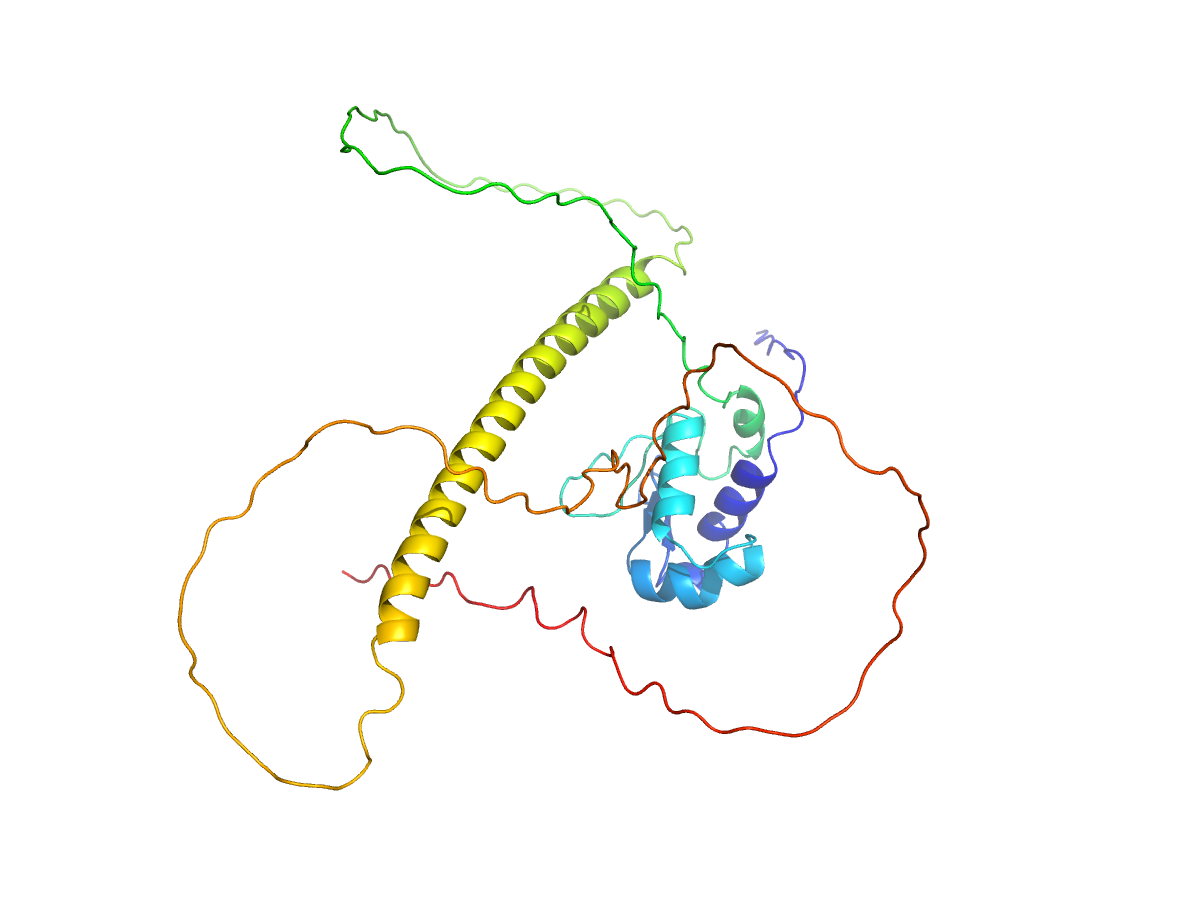


CeHSF15:


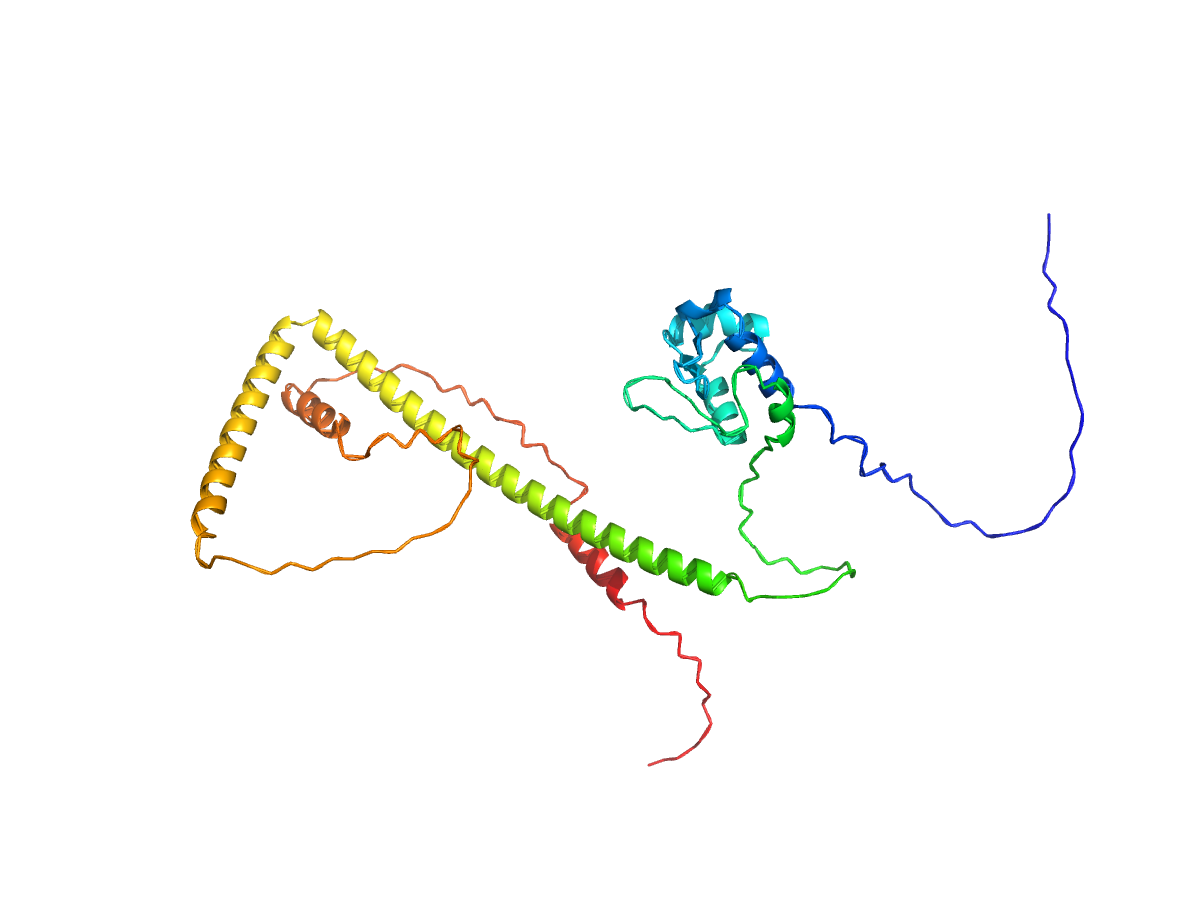


CeHSF16:


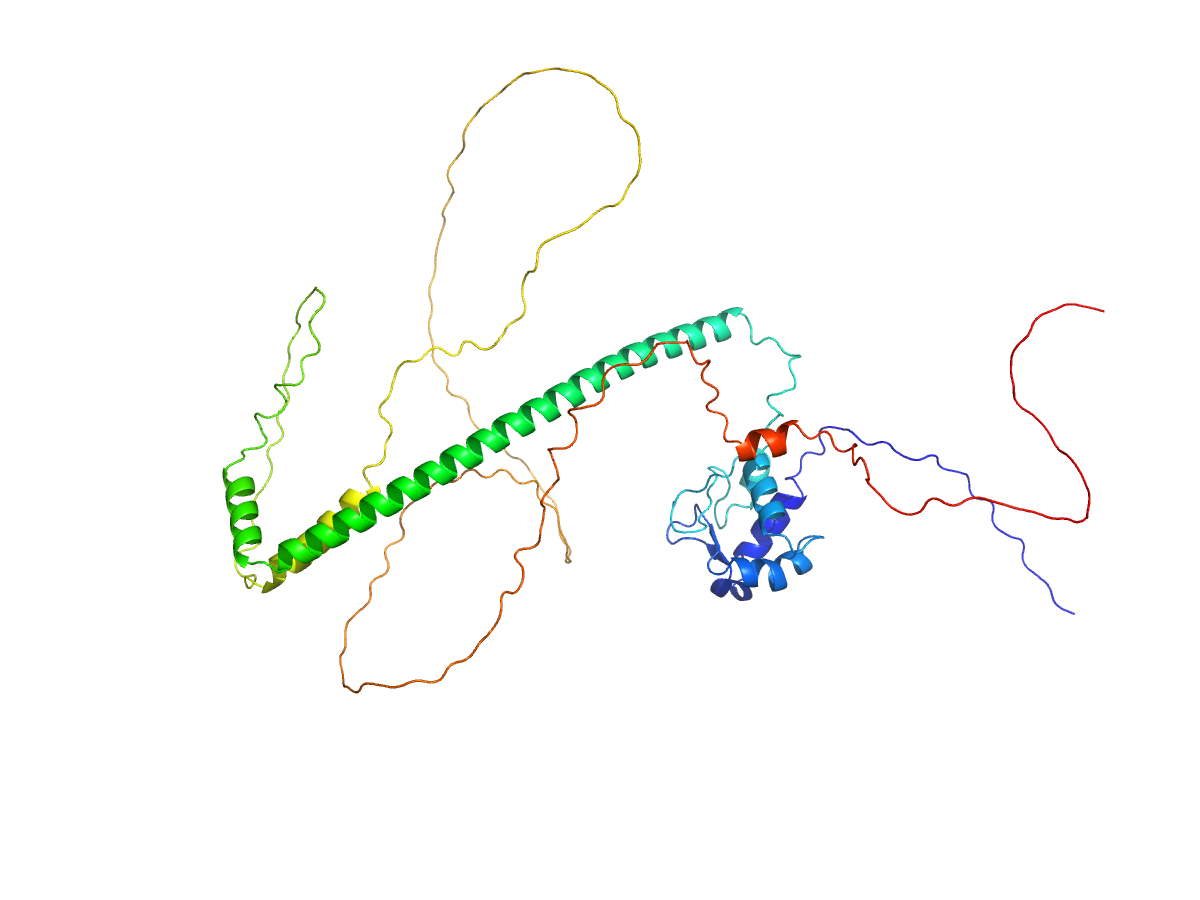


CeHSF17:


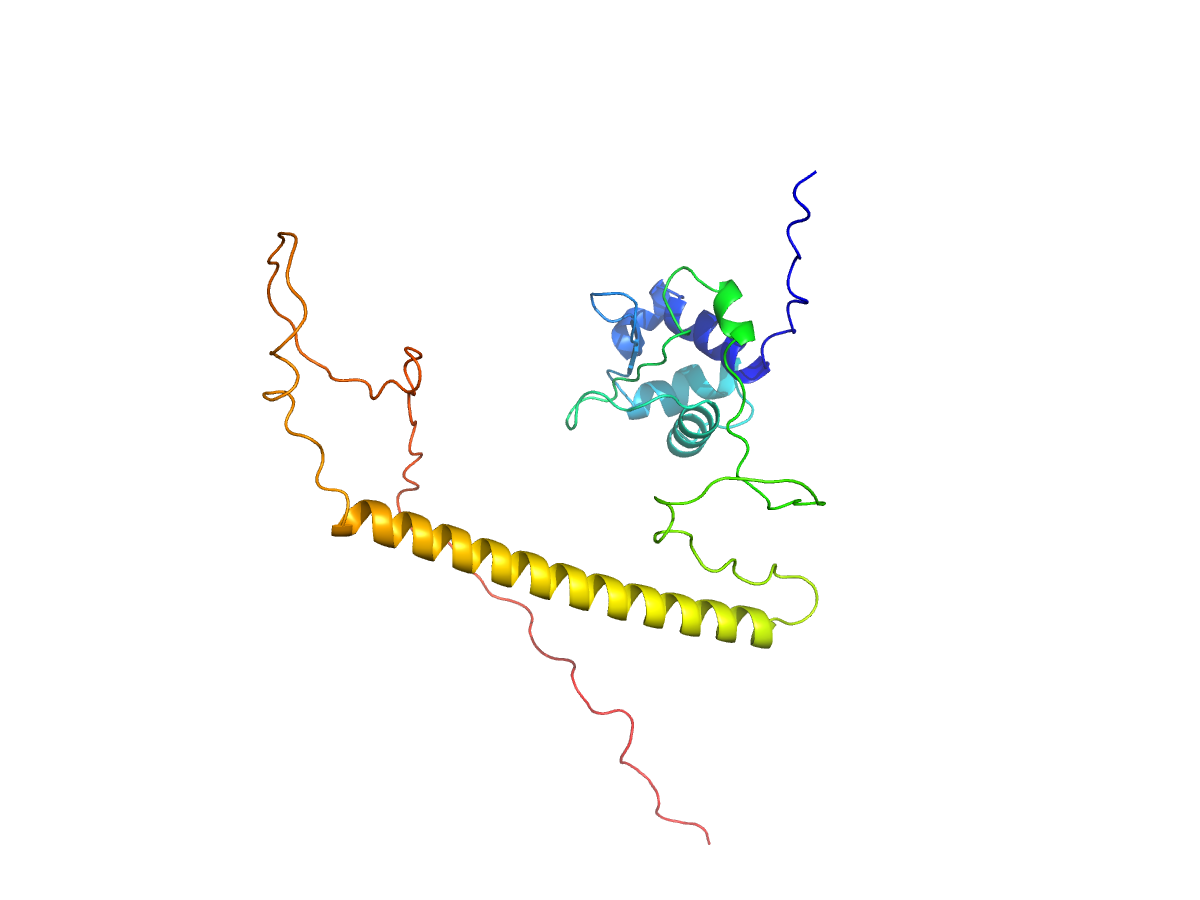


CeHSF18:


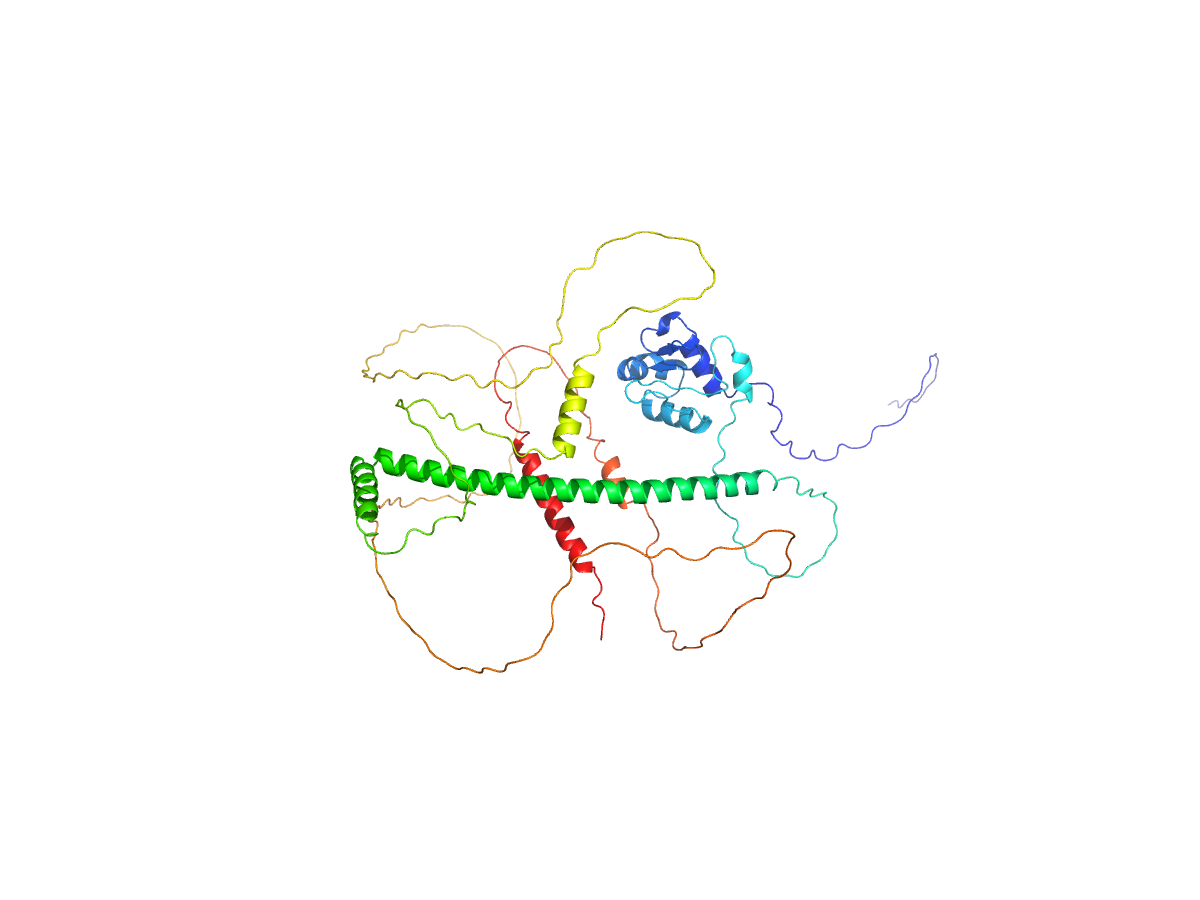


CeHSF19:


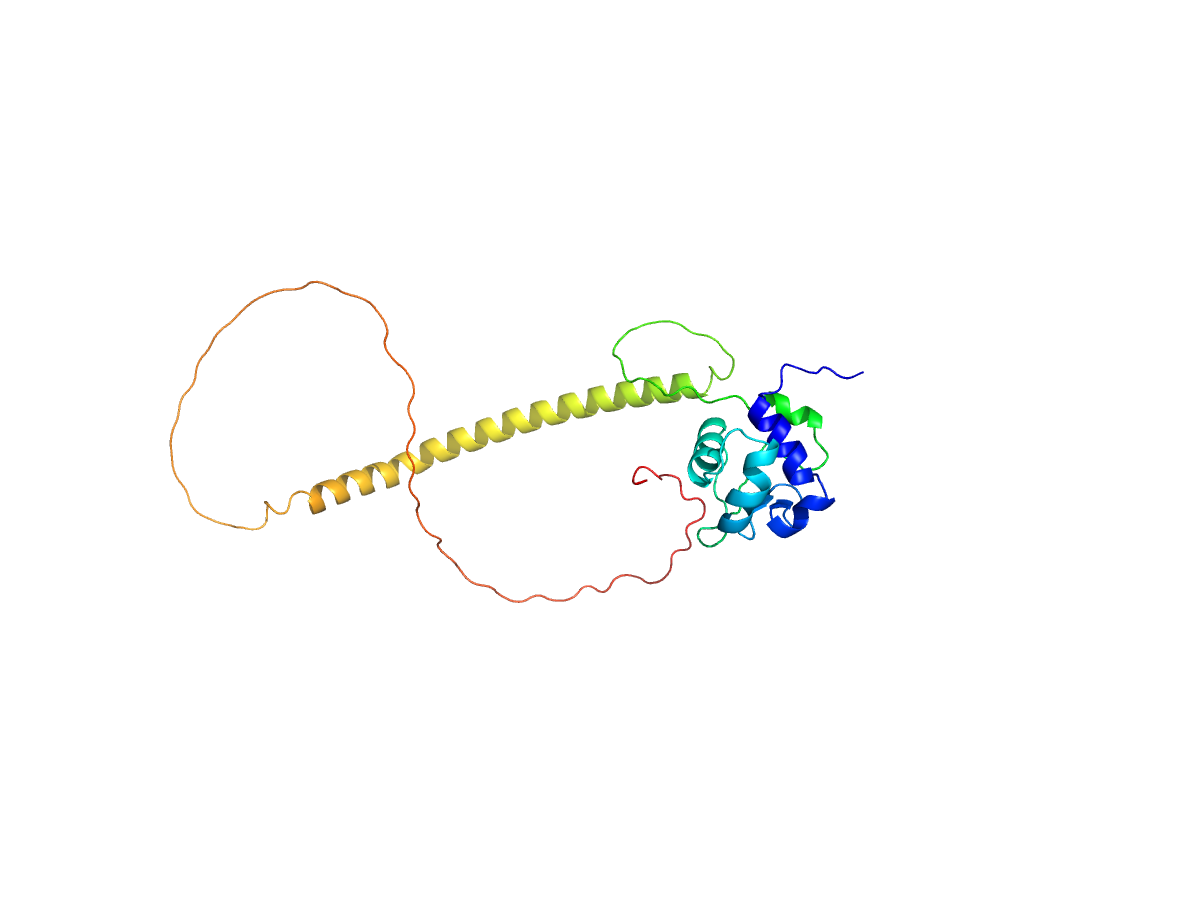


CeHSF20:


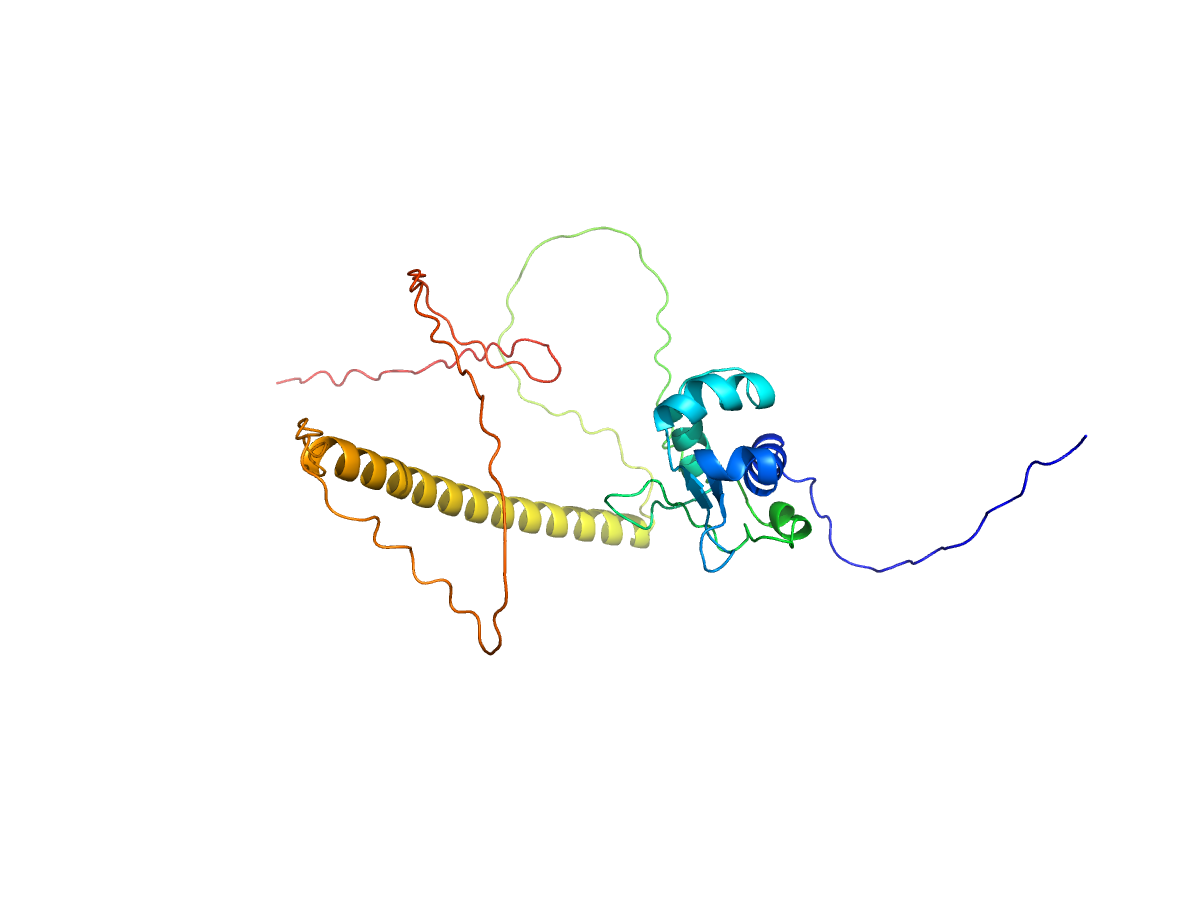


CeHSF21:


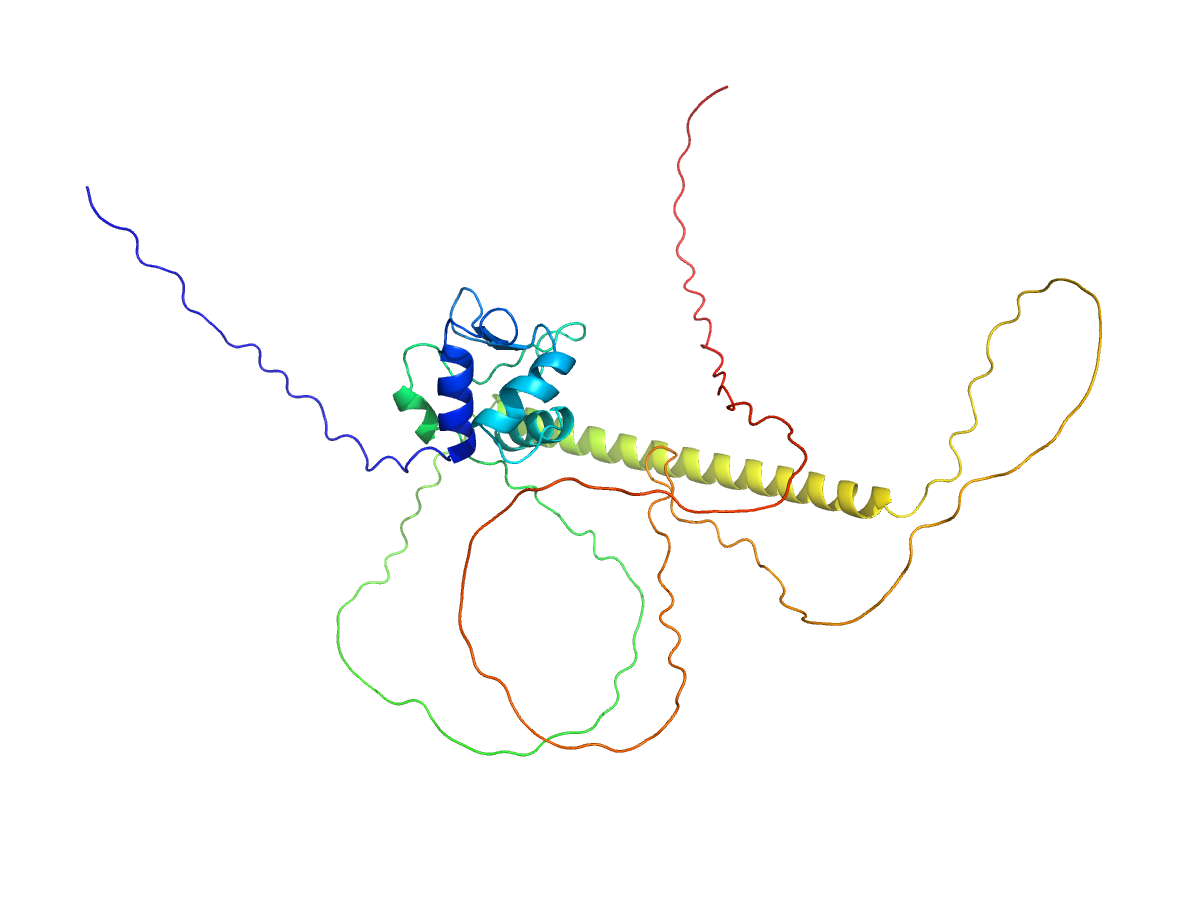


CeHSF22:


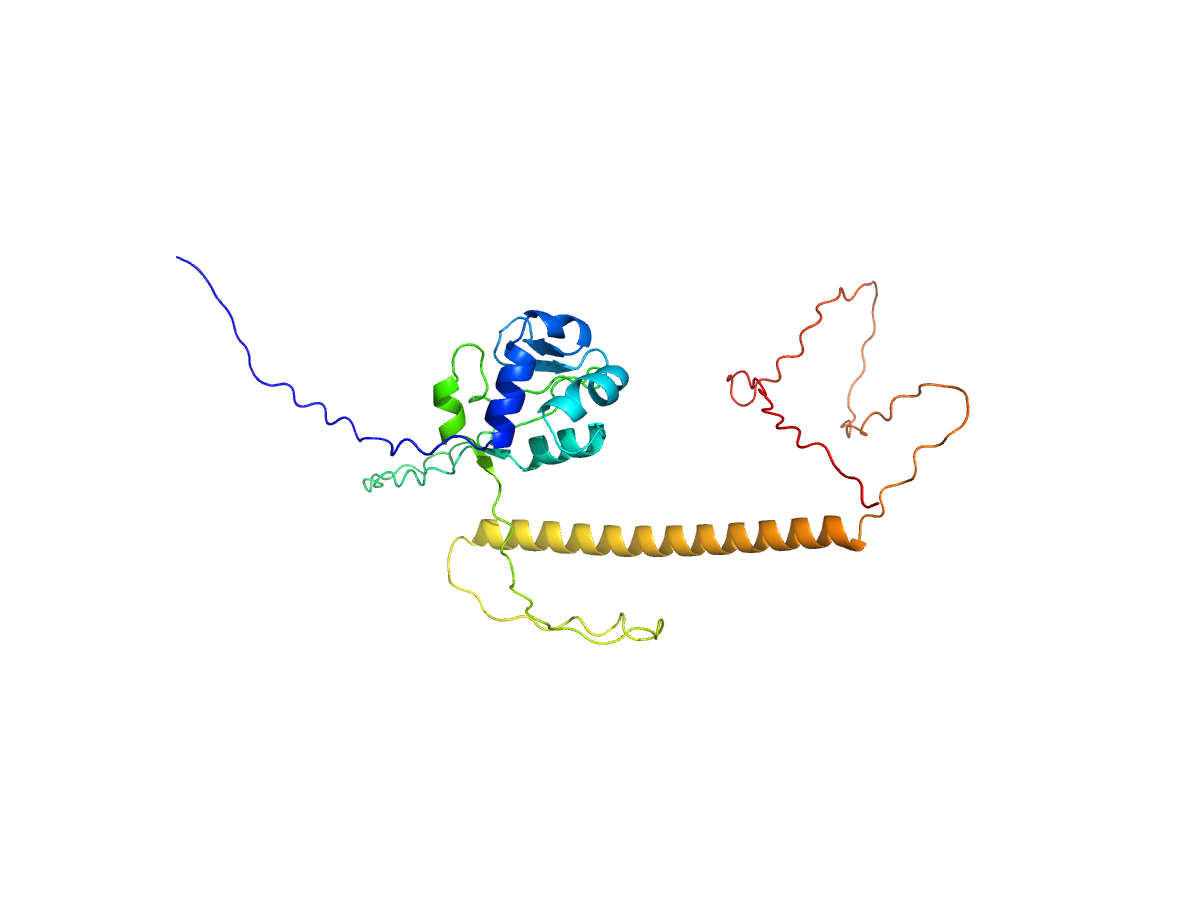

Supplement: Supplementary file 1 [file ijms-25-01002-s001.zip › Supplementary Table S2.docx]
